# Antifluorite-derived Li_7_MnN_4_: revisiting the crystal structure and catalysis in ammonia decomposition

**DOI:** 10.1039/d5cy01547b

**Published:** 2026-03-09

**Authors:** Mirabbos Hojamberdiev, Ana Laura Larralde, Eva M. Heppke, Oscar Gómez-Cápiro, John Carl A. Camayang, Thomas Bredow, Kunio Yubuta, Katsuya Teshima, Tamanna M. Ahamad, Christian Lorent, Liqun Kang, Yves Kayser, Holger Ruland, Serena DeBeer, Martin Lerch

**Affiliations:** a Institut für Chemie, Technische Universität Berlin Straße des 17. Juni 135 10623 Berlin Germany hmirabbos@gmail.com; b Mads Clausen Institute, University of Southern Denmark Alsion 2 6400 Sønderborg Denmark mirabbos@mci.sdu.dk; c Consejo Nacional de Investigaciones Científicas y Técnicas (CONICET) Buenos Aires Argentina; d Instituto Nacional de Tecnología Industrial Avenida General Paz 5445 San Martín (B1650WAB) Buenos Aires Argentina; e Department of Heterogeneous Reactions, Max Planck Institute for Chemical Energy Conversion Stiftstraße 34–36 45470 Mülheim an der Ruhr Germany; f Department of Inorganic Spectroscopy, Max Planck Institute for Chemical Energy Conversion Stiftstraße 34–36 45470 Mülheim an der Ruhr Germany; g Mulliken Center for Theoretical Chemistry, Clausius-Institut für Physikalische und Theoretische Chemie, University of Bonn Beringstraße 4 53115 Bonn Germany; h Institute for Aqua Regeneration, Shinshu University 4-17-1 Wakasato Nagano 380-8553 Japan

## Abstract

Catalytic ammonia decomposition is a sustainable chemical route for hydrogen production. Transition metal nitrides have emerged as promising and effective catalysts for this reaction. In this study, we revisit the synthesis, crystal structure, optoelectronic properties, and catalytic performance of antifluorite-derived Li_7_MnN_4_. Phase-pure Li_7_MnN_4_ powder is synthesized from Li_3_N and metallic Mn at 800 °C in a tantalum ampoule, resulting in a highly crystalline cubic phase with space group *P*4̄_3_*n* (no. 218), a lattice parameter of *a* = 9.5598(8) Å, and a unit cell volume of 873.66(14) Å^3^. Rietveld refinement results show excellent residual factors (*R*_wp_ = 1.71, *S* = 1.38), confirming the ordered arrangement of [MnN_4_]^7−^ tetrahedra and five symmetrically distinct Li sites. The experimental data are complemented by density functional theory calculations, revealing weak spin coupling consistent with a paramagnetic ground state. Strong absorption in the UV-visible region corresponds to an experimental optical band gap of ∼2.76 eV, while Raman and infrared spectra are dominated by MnN_4_ tetrahedral vibrations. X-ray absorption spectroscopy indicates a high Mn oxidation state and a well-defined Mn–N/Li coordination. Catalytic tests show that Li_7_MnN_4_ and Li_7_MnN_4_ : LiNH_2_ (1 : 1 molar ratio) exhibit activities comparable to a Ni-based reference catalyst, with apparent activation energies of 364.4 kJ mol^−1^ and 256.0 kJ mol^−1^, respectively, highlighting the beneficial effect of LiNH_2_ incorporation. Thermogravimetry coupled with mass spectrometry identifies decomposition pathways involving LiNH_2_/Li_2_NH intermediates and forming Li_3_N and manganese nitrides. These results demonstrate that Li_7_MnN_4_ is a catalytically promising nitride for ammonia decomposition, with potential for further optimization through compositional tuning and mechanistic insights.

## Abstract 2

Antifluorite-derived Li_7_MnN_4_ was synthesized, and its ordered crystal structure was resolved. Ammonia decomposition proceeds *via* lithium amide-imide-nitride transformations, enabling its catalytic performance comparable to Ni-based catalysts.

## Graphical abstract

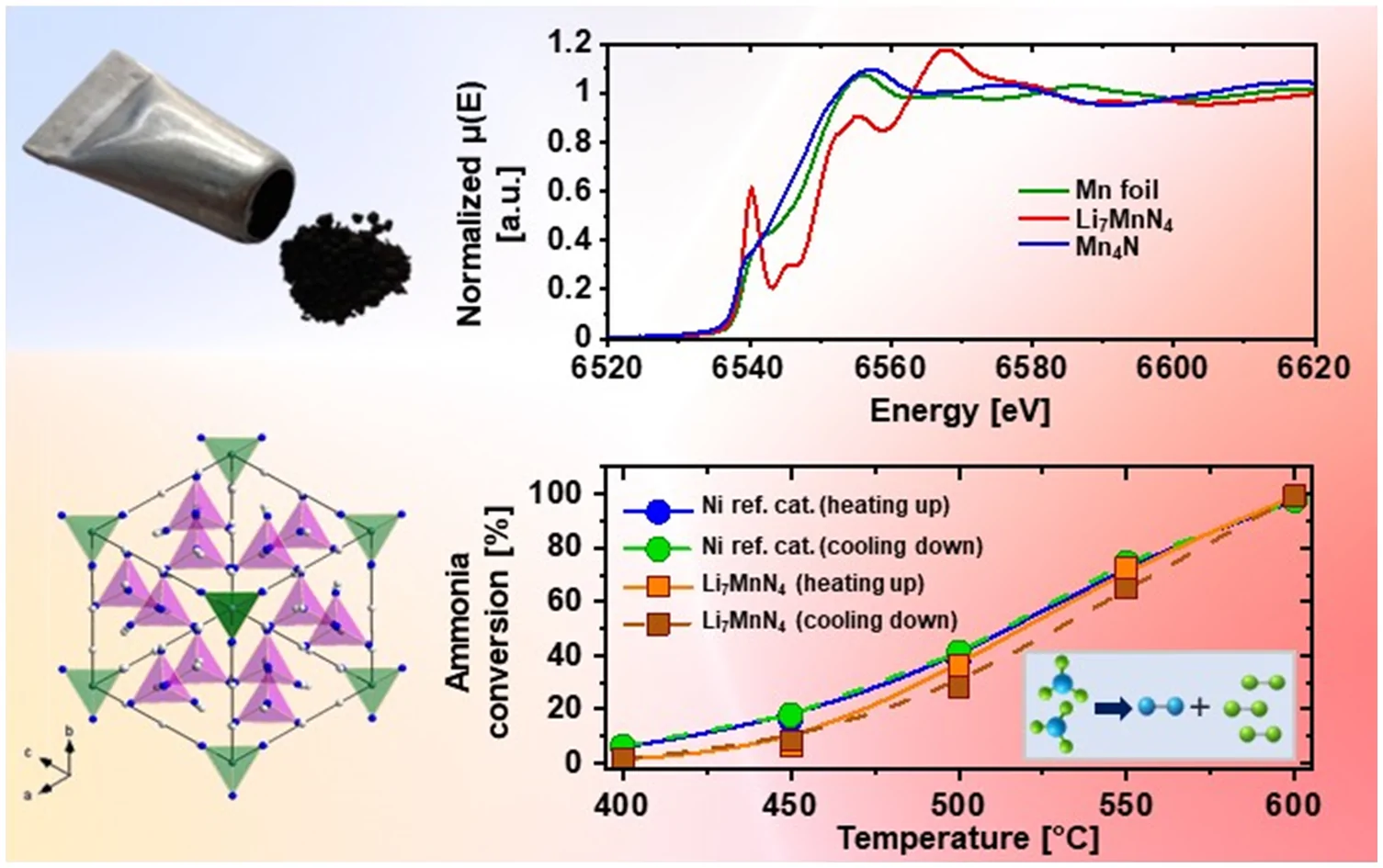

## 1. Introduction

Ammonia is an efficient compound for hydrogen storage and transportation due to its high volumetric hydrogen density (123 kg m^−3^), high gravimetric hydrogen density (17.7%), and well-developed technologies for its production, liquefaction, storage, and transportation.^1,2^ However, to enable its use in fuel-cell vehicles, ammonia must be catalytically decomposed into hydrogen and nitrogen at a relatively high temperature.^3,4^ Therefore, a rational catalyst design is crucial for advancing ammonia as a clean energy source.^5^ Various binary and ternary transition metal nitrides have been explored as inexpensive, efficient, and stable catalyst alternatives to noble metal-based catalysts (*e.g.*, Ru) for ammonia decomposition.^6,7^ For instance, the MnN–Li_2_NH composite demonstrated superior catalytic activity for hydrogen production compared to the highly active 5 wt% Ru/CNTs catalyst during ammonia decomposition.^8^ This enhancement was linked to the alteration of reaction pathways and energetics, driven by the inductive effect of Li^+^ on the covalent Mn–N bond. Hund *et al.*^9,10^ investigated the thermal properties of Fe_3_Mo_3_N and Ni_2_Mo_3_N in an NH_3_ atmosphere and their catalytic performance in ammonia decomposition. Their findings revealed that, in contrast to Ni_2_Mo_3_N, Fe_3_Mo_3_N underwent decomposition at elevated temperatures, forming binary nitrides (MoN and Fe_2_N), facilitating the ammonia decomposition process. Recently, the synthesis, crystal structure, and stability of ordered antifluorite-derived Li_14_Cr_2_N_8_O were revisited, and its catalytic activity was evaluated as the first quaternary transition metal nitride oxide and its potential as a promising catalyst for ammonia decomposition.^11^ Mambretti *et al.*^12^ explored the process of ammonia decomposition on Li_14_Cr_2_N_8_O using machine learning-enabled molecular dynamics simulations and found evidence that Li_14_Cr_2_N_8_O is strongly altered during ammonia decomposition with enhanced movement of lithium atoms and formation of an ad-layer of amides. The structural, thermal, and catalytic activity for ammonia decomposition of Li_2_ZrN_2_ were recently studied.^13^ According to *in situ* XRD and TG-MS data, the structural instability of Li_2_ZrN_2_ could influence its catalytic activity, with ammonia decomposition beginning above 500 °C and improving with repeated heating–cooling cycles, likely due to the formation of active sites.

Within the class of lithium-based transition metal nitrides that adopt an antifluorite-derived crystal structure, Li_7_MnN_4_ has garnered considerable interest. Li_7_MnN_4_ was first synthesized by Juza *et al.*^14^ in the late 1950s. Its cubic symmetry with space group *P*4̄_3_*n* was validated by Niewa *et al.*^15^ using X-ray diffraction data for a single crystal. Later, the antifluorite-derived structure of Li_7_MnN_4_ was confirmed by Cabana *et al.*^16^ using powder neutron diffraction data. Li_7_MnN_4_ has been extensively explored as a promising electrode material for Li-ion battery due to its operation voltage of 1.2 V, larger specific capacity of 250 mAh g^−1^ at 1 C rate, and excellent cycling performance (96% capacity retention at C rate after 100 cycles) because of its small and highly reversible breathing during the redox process.^17–19^ Nishijima *et al.*^20^ first studied a lithium deintercalation-intercalation process in Li_7_MnN_4_, revealing its good reversibility and high current density (1200 μA cm^−2^). A study on the electronic structure and the charge balance mechanism using core-level electron energy loss spectroscopy classified Li_7_MnN_4_ into the charge-transfer regime.^21^

Li_7_MnN_4_ is highly sensitive to moisture and reactive in air and undergoes degradation for a prolonged time *via* an acid–base reaction with moisture, first releasing a small amount of lithium and resulting in the formation of Li_6.2_MnN_4_, and then, leading to a phase transition to lithium hydroxide and lithium carbonate with the generation of ammonia gas.^22^ Using Raman spectroscopy, Zhou *et al.*^19^ effectively monitored the change in the manganese oxidation state and reactivity of Li_7_MnN_4_, indicating its complete degradation after 100 min of air exposure. Small polaron hopping conduction with an activation energy of 112 meV was observed at >180 K, and a transition from hopping to tunneling conduction occurred at <60 K for Li_7_MnN_4_, suggesting small polaron motion in the Mn-3d band.^23^ The paramagnetic behavior of Li_7_MnN_4_ was observed due to the Mn-3d electrons partially occupying the Mn-3d band, and the optical absorption edge was calculated to be 1.18 eV.^24^ He *et al.*^25^ investigated the hydrogen storage property of Li_7_MnN_4_ and concluded that, despite the absorption of 7 hydrogen atoms per unit of Li_7_MnN_4_, its practical use in fuel cell vehicles is hindered by the release of NH_3_ and the relatively high absorption temperature.

Due to the formation of Li_2_NH, which favors the formation of a higher N-content intermediate during the ammonia decomposition reaction^8,26^ and the rich redox chemistry, low-cost, earth-abundance, and non-toxicity of manganese,^27^ the compounds in the Li–Mn–N system are of particular interest in the catalytic ammonia decomposition. Therefore, this research emphasizes the synthesis, crystal structure analysis, and computational investigation of various properties at the density-functional theory (DFT) level of antifluorite-derived Li_7_MnN_4_. Also, this study explores the catalytic activity of antifluorite-derived Li_7_MnN_4_ for ammonia decomposition.

## 2. Experimental

### 2.1. Synthesis

The antifluorite-derived Li_7_MnN_4_ phase was synthesized in powder form by a solid-state reaction. Li_3_N (99.4%, Thermo Scientific) and metallic Mn (99.98%, chemPUR) were manually mixed in a 7 : 1 proportion using an agate mortar and pestle and placed into a tantalum ampoule measuring 5.5 cm in length and 1.4 cm in diameter in an Ar-filled glovebox. The ampoule containing the powder mixture was manually sealed using a bench vice and subjected to heat treatment on a corundum boat in a tube furnace at 800 °C for 20 h, with a heating rate of 180 K h^−1^ and a cooling rate of 300 K h^−1^, under nitrogen gas flow (10 L h^−1^). The sample was collected, homogenized, and subjected to various chemical and structural characterizations.

### 2.2. Characterization

The X-ray diffraction (XRD) pattern was acquired using a PANalytical X‘Pert Pro powder diffractometer operated with nickel-filtered Cu-Kα radiation at 40 kV and 30 mA. The powder diffraction data were collected in a Bragg–Brentano setup with a *θ*/*θ*-arrangement at ambient temperature over an angular range of 2*θ* = 10–120° with a step size of 0.026°. Rietveld refinements^28^ were executed with the program package FULLPROF SUITE 2021 (ref. 29) by applying a pseudo-Voigt function. The nano- and microstructures were examined using transmission electron microscopy operated at 160 kV (EM-002B, TOPCON) and scanning electron microscopy operated at 4 kV (SEM; Zeiss Gemini 982, Carl Zeiss), respectively. The UV-vis diffuse reflectance spectrum was measured using an Evolution 220 UV/Vis spectrometer (Thermo Fisher Scientific).

Resonance Raman spectroscopy was performed using the 488 nm line of an Ar^+^ laser (Coherent) attenuated to 0.5 mW power on the sample. Measurements were carried out at 80 K with a liquid-nitrogen-cooled cryostat (Linkam Scientific Instruments). The spectrum was acquired with a LabRam HR-800 confocal Raman spectrometer (Jobin Yvon) equipped with a liquid-nitrogen-cooled CCD detector and represents an average of 30 scans with an acquisition time of 150 s. Toluene served as an external reference for frequency calibration. Data analysis was performed using Bruker OPUS software (version 6.5 or later).

The X-ray absorption fine structure (XAFS) spectra of the samples were collected at BM31 of the Swiss-Norwegian Beamlines (SNBL) at the European Synchrotron Radiation Facility (ESRF), Grenoble, France.^30^ The Mn-based nitride powders were homogeneously mixed with BN diluent at composition-dependent mass ratios and loaded into quartz capillaries (1 mm OD, 10 μm wall thickness; WJM Glas, Müller GmbH). Each loaded capillary tube was then mounted in an air-tight holder to prevent exposure to the ambient atmosphere during the XAFS measurements. All sample handling and loading were carried out in an Ar-filled glovebox due to the high air- and moisture-sensitivity of Li_7_MnN_4_ and Mn_4_N. Further details on instrumentation, data acquisition, and processing are provided in the SI (Section 1).

### 2.3. Catalytic activity test on ammonia decomposition and apparent activation energy

The ammonia decomposition activity of the materials was evaluated and compared to an industrial Ni-based catalyst under identical conditions. Before the reaction, the samples were pressed at up to 5 tons for 3 min to form pellets, which were subsequently ground and sieved to obtain particles of 200–300 μm.

All sample preparation steps were carried out in an Ar-filled glovebox. For catalytic tests, 200 mg of sample was diluted with SiC (300–400 μm) in a 1 : 2 mass ratio and loaded into a quartz tubular reactor (inner diameter: 5.25 mm). The reaction was carried out under a constant flow of ammonia (120 mL min^−1^, 5.0 purity), which was passed through an activated metal-based adsorption filter (MicroTorr GateKeeper GPUS) to remove trace contaminants. The test temperature was ramped from 400 °C to 600 °C in 50 °C increments and then decreased to 400 °C. Each temperature step was held for 60 min, with a heating/cooling rate of 2.0 K min^−1^. Prior to switching to ammonia, the catalyst bed was preheated to 400 °C under a constant flow of N_2_ (200 mL min^−1^, filtered) at a rate of 0.8 K min^−1^, and held at 400 °C for 1 h to ensure thermal equilibration.

A second set of experiments was conducted using ammonia, bypassing the filter to assess the impact of potential impurities. For the determination of the apparent activation energy, 27.3 mg of catalyst was homogeneously mixed with 400 mg of SiC and tested under the same initial screening conditions (120 mL min^−1^ filtered ammonia, 50 °C temperature steps). After screening and starting from 400 °C, the ammonia stream was diluted to 50 vol% with filtered helium (thermal conductivity of helium is 0.15 W m^−1^ K^−1^*vs.* 0.023 W m^−1^ K^−1^ for ammonia) to reduce the thermal effect of the endothermic reaction. The temperature was then increased from 400 °C to 600 °C in 10 °C increments, with each step held for 90 min. The heating rate between steps was maintained at 2.0 K min^−1^. Apparent activation energies were estimated from the Arrhenius plots, using only data points with ammonia conversion below 10%. Reaction effluents were analyzed using an Emerson XStream XEGP gas analyzer equipped with infrared (IR) detectors for NH_3_ and H_2_O, and a thermal conductivity detector (TCD) for H_2_. The data was collected with a time resolution of 1 min. For the calculation of mean conversion and reaction rates, only the last 30 values of ammonia concentration at each temperature step were used. Further details of the experimental setup and test protocols were previously described elsewhere by Gómez-Cápiro *et al.*^31^

The Li_7_MnN_4_ sample was also tested in a thermogravimetry-mass spectrometry (TG-MS) setup under ammonia decomposition conditions. A gas flow of 30 mL min^−1^ of ammonia was introduced into the TG oven, with an additional 60 mL min^−1^ of helium as a protective gas. The powder sample was placed in a pre-weighed alumina crucible at the center of the oven. All sample preparation was carried out in an Ar-filled glovebox prior to transfer to the TG device (PerkinElmer TGA 8000). The temperature program started with a heating rate of 3 K min^−1^ until 400 °C, followed by a 30 min hold. Temperature was then increased in 50 °C increments at 2.5 K min^−1^, with 30 min holds at each step, until 600 °C was reached. After holding at 600 °C for 30 min, the final segment was conducted at 550 °C for 120 min. Evolved gases were analyzed by mass spectrometry (PerkinElmer Clarus® SQ 8 T), monitoring *m*/*z* 17 (ammonia) and *m*/*z* 28 (nitrogen) to follow ammonia decomposition.

## 3. Results and discussion

Li_7_MnN_4_ was first synthesized and determined by Juza *et al.*^14^ in 1959 to crystallize in a distorted antifluorite-derived superstructure. Niewa *et al.*^15^ synthesized single crystals of Li_7_MnN_4_ from reactions of Li and Mn with nitrogen gas in the presence of Ba at 1073 K, along with Li_3_N and Ba_3_MnN_3_, and re-determined its crystal structure from the X-ray diffraction data. Later, Cabana *et al.*^16^ prepared pure Li_7_MnN_4_ from a powder mixture of Li_3_N and Mn_*x*_N (a commercial mixture of Mn_4_N and Mn_2_N) by pelletizing and thermally treating at 650 °C for 6 h under nitrogen flow and confirmed its cubic ordered antifluorite-derived structure using powder neutron diffraction data. Also, Zhou *et al.*^19^ synthesized Li_7_MnN_4_ by a solid-state reaction of the pellets of the Li_3_N and Mn mixture at 750 °C for 12 h under a N_2_ flow, and their Rietveld refinement data are in good agreement with those of Cabana *et al.*^16^ In this study, phase-pure Li_7_MnN_4_ was synthesized in powder form from Li_3_N and metallic Mn (7 : 1 ratio) in a tantalum ampoule at 800 °C for 20 h, without pelletizing and using expensive or highly reactive starting materials, providing a simpler synthetic route.

**Fig. 1:**
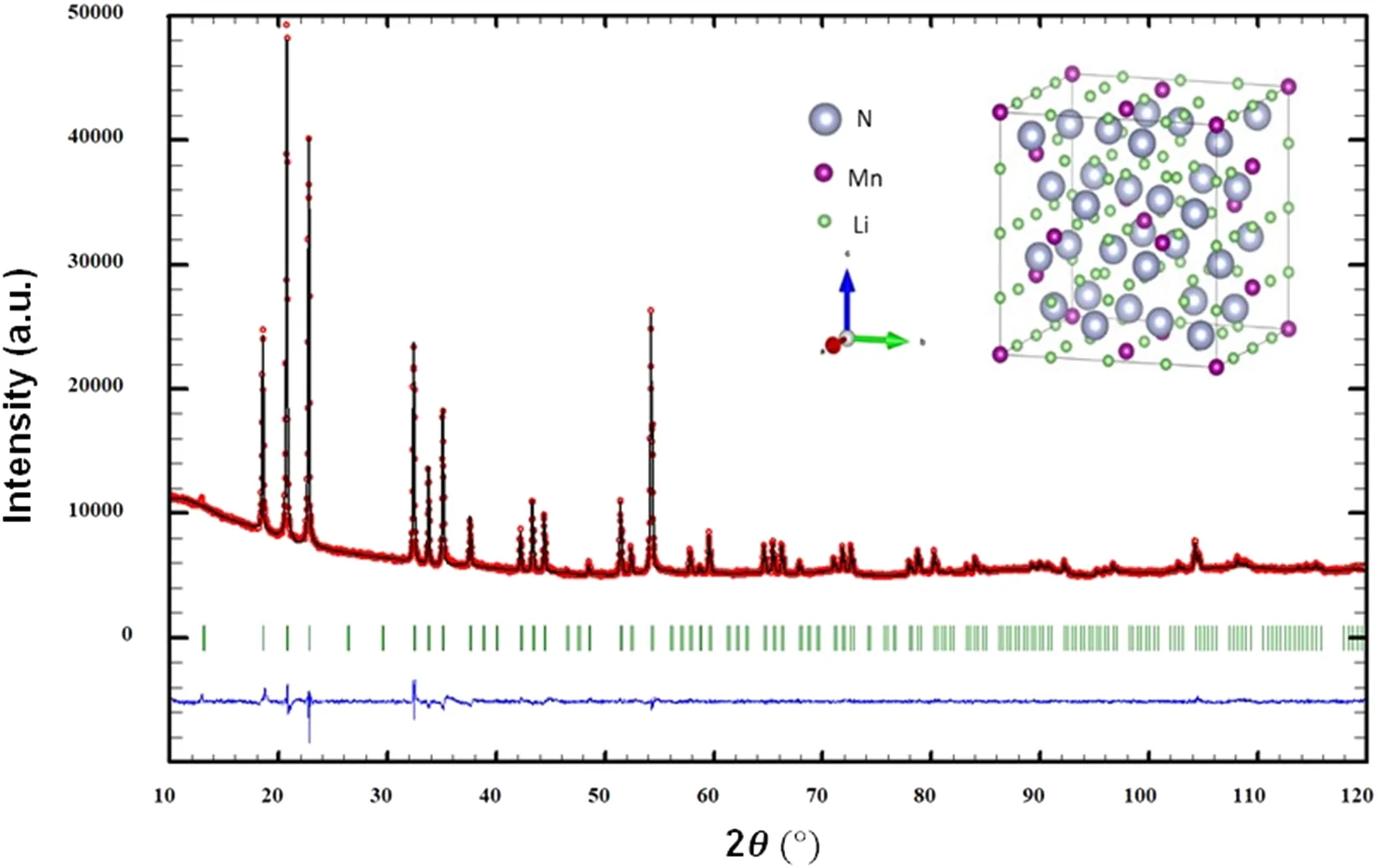
X-ray diffraction patterns of Li_7_MnN_4_ powder with the results of the Rietveld refinement. The measured pattern, calculated pattern, difference plot, and calculated Bragg reflections are represented in red circles, black solid line, blue solid line, and green tick marks, respectively.

The sharp reflections in the XRD pattern of the synthesized Li_7_MnN_4_ indicate its high crystallinity. The powder X-ray diffraction data were refined with the program FULLPROF SUITE 2021 (ref. 29) using the model proposed by Cabana *et al.*^16^ as an input structure, which is available through the joint CCDC/FIZ Karlsruhe online deposition service (https://www.ccdc.cam.ac.uk/structures/) under deposition number CSD-1670745. The coordinates of the Li positions originate from the quantum-chemical calculations we used and kept fixed during refinement. As shown in Fig. 1, a good refinement quality was achieved with reasonable residual values (*R*_wp_ = 1.71 and *S* = 1.38). No reflections indicating the presence of impurity phases were detected; the deviations at small angles may point to disorder. Li_7_MnN_4_ crystallizes isotypically to Li_7_PN_4_ in a distorted 2 × 2 × 2 antifluorite-derived superstructure in space group *P*4̄_3_*n* (no. 218) with a unit-cell parameter *a* = 9.5598(8) Å, which is consistent with the reported values.^16,19^

**Fig. 2:**
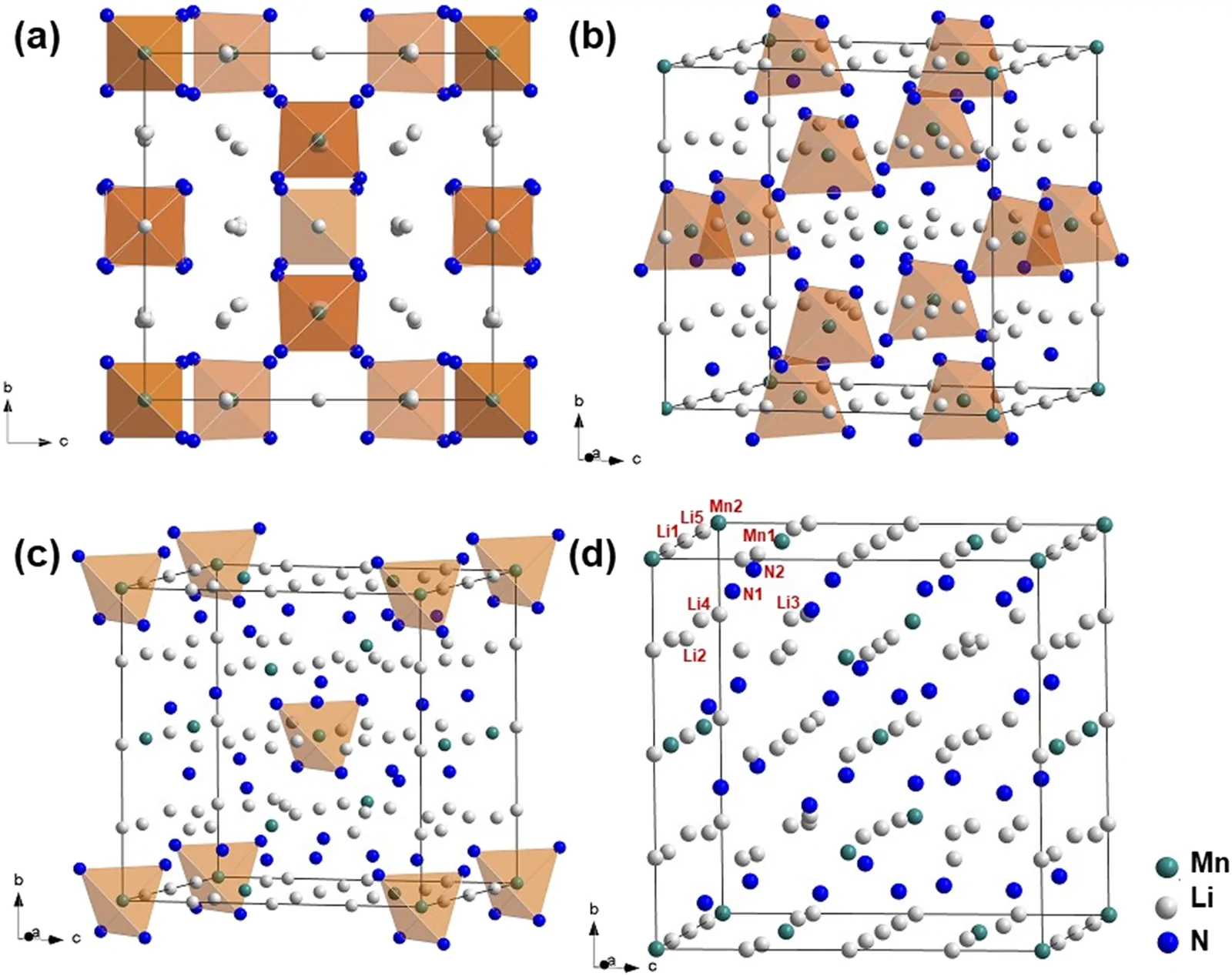
Unit cell of Li_7_MnN_4_ with an indication of (a) all isolated [MnN_4_]^7−^ tetrahedra, (b) [Mn1N1_4_]^7−^ tetrahedra, (c) [Mn2N2_4_]^7−^ tetrahedra, and (d) atoms in the unit cell.

The refined parameters and residual factors are given in Tables 1 and 2. In the primitive cubic unit cell, the Mn1, Mn2, N1, and N2 atoms occupy 6*c*, 2*a*, 24*i*, and 8*e* Wyckoff positions, respectively (Table 2). There are five symmetrically nonequivalent Li atoms in the unit cell (Table 2). Two of them (Li1 and Li2) are located in high symmetry 6*b* and 6*d* Wyckoff positions, respectively. The two others (Li3 and Li5) are situated in low symmetry 8*e* and 12*f* Wyckoff positions, respectively, and the last Li atom (Li4) occupies a general 24*i* Wyckoff position. As shown in Fig. 2, the crystal structure of Li_7_MnN_4_ is built of [MnN_4_]^7−^ tetrahedra, which are stacked together with the Li atoms in a CaF_2_ anti-type arrangement. The crystal structure of Li_7_MnN_4_ features two types of isolated [MnN_4_]^7−^ tetrahedra: (i) Mn1 atoms, which occupy low-symmetry sites, are bonded to four N1 atoms (low symmetry), and (ii) Mn2 atoms, which are located at the vertices and body center of the cubic unit cell, are bonded to four N2 atoms (high symmetry). Lithium atoms are coordinated tetrahedrally by nitrogen, which is surrounded by a distorted cube built of one Mn and seven Li.^15^ In Tables S1, S2, and S6, the interatomic distances and bond angles are compared with the powder neutron diffraction data and single crystal X-ray diffraction data.^15,16^ It was found that the interatomic distances are in good agreement with previous studies. The bond angles confirm the cubic and tetrahedral coordination of anions and cations, respectively.

**Table 1:** Results of the Rietveld refinement for Li_7_MnN_4_ (standard deviations in parentheses)

Empirical formula	Li_7_MnN_4_
Structure type	Li_7_PN_4_
Space group	*P*4̄_3_*n* (no. 218)
Crystal system	Cubic
*Z*	8
*a* (Å)	9.5598(8)
*V* (Å^3^)	873.66(14)
Calculated density (g cm^−3^)	2.426
Diffractometer	PANalytical X'Pert Pro
Radiation	Cu-Kα
Wavelength (Å)	*λ*_1_ = 1.54056, *λ*_2_ = 1.54439
*R*_p_	1.21
*R*_wp_	1.71
*R*_exp_	1.25
*R*_Bragg_	4.93
*S*	1.38

**Table 2:** Refined atomic parameters for Li_7_MnN_4_ (standard deviations in parentheses)

Atom	Wyckoff	*x*	*y*	*z*	s.o.f.	*B*_iso_/Å^2^
Li1	6*b*	0	1/2	1/2	1	1
Li2	6*d*	1/4	0	1/2	1	1
Li3	8*e*	0.2360	0.2360	0.2360	1	1
Li4	24*i*	0.2497	0.2384	−0.0175	1	1
Li5	12*f*	0.2640	0	0	1	1
Mn1	6*c*	1/2	0	1/4	1	0.77(11)
Mn2	2*a*	0	0	0	1	0.45(19)
N1	24*i*	0.3607(5)	0.3821(8)	0.1097(19)	1	1
N2	8*e*	0.1079(19)	0.1079(19)	0.1079(19)	1	1

**Fig. 3:**
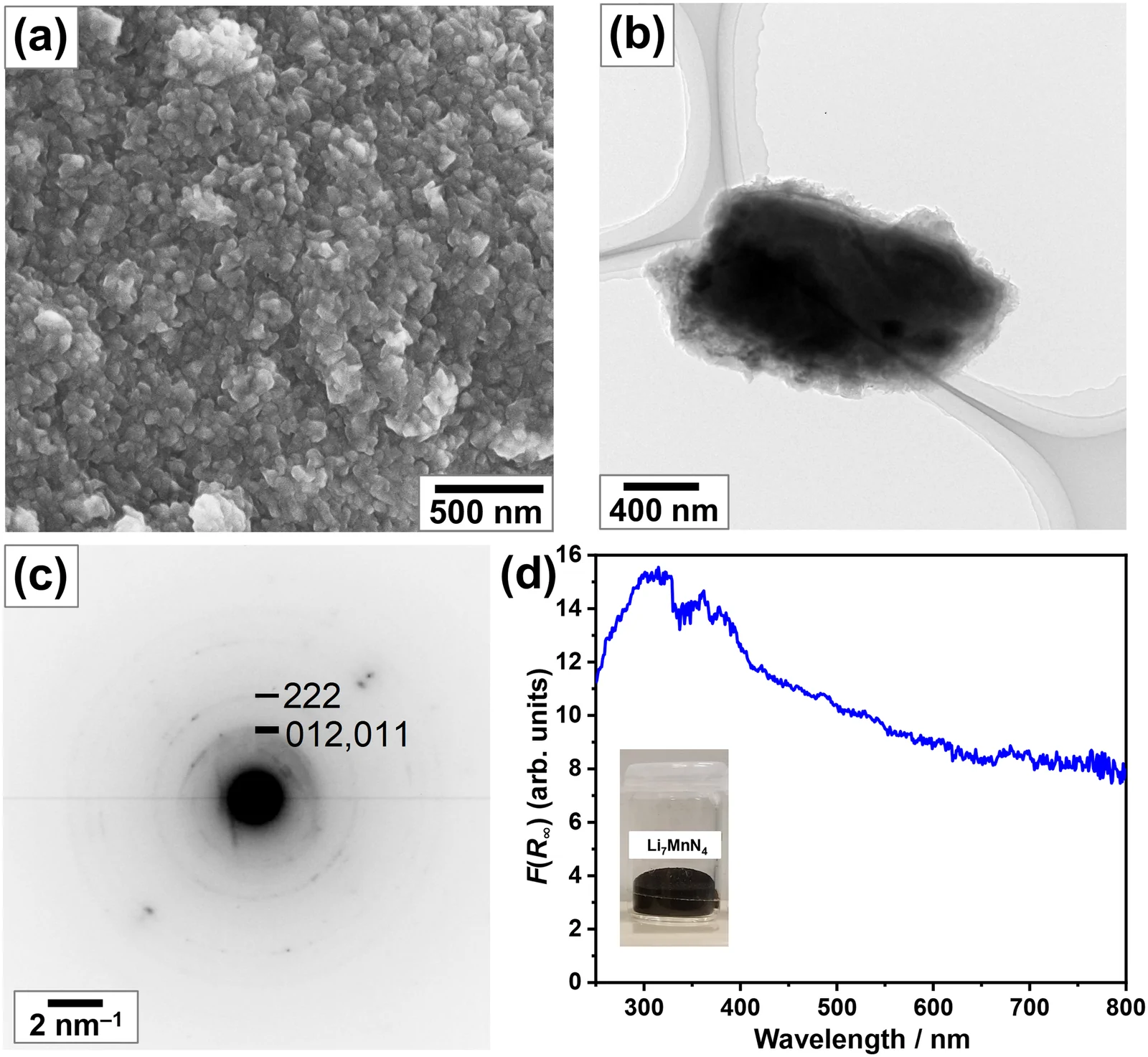
(a) SEM image, (b) TEM image, (c) SAED pattern, and (d) UV-vis diffuse reflectance spectrum of Li_7_MnN_4_ powder.

The surface morphology of Li_7_MnN_4_ powder was examined using scanning electron microscopy (SEM). In Fig. 3a, the SEM image shows an aggregated microstructure composed of nanocrystalline grains with a relatively rough surface. The observed granular texture suggests the formation of polycrystalline particles, which may influence its optoelectronic properties, reactivity, and catalytic activity. Fig. 3b shows the transmission electron microscopy (TEM) image of a Li_7_MnN_4_ particle, revealing an irregular morphology. The particle exhibits high density, indicative of its highly crystalline nature. Note that the difference between the SEM and TEM images of Li_7_MnN_4_ particles arises because SEM shows agglomerated particles, whereas TEM resolves the smaller primary crystallites. The crystallinity of Li_7_MnN_4_ powder was further investigated through selected area electron diffraction (SAED) analysis. In Fig. 3c, the diffraction rings correspond to the indexed planes of Li_7_MnN_4_, including the (222) and (012, 011) reflections, confirming its polycrystalline nature. The presence of distinct diffraction spots suggests a well-ordered structure.

The optical properties of Li_7_MnN_4_ powder were investigated using UV-vis diffuse reflectance spectroscopy (DRS), and the corresponding spectrum is shown in Fig. 3d. The Li_7_MnN_4_ powder exhibits strong absorption throughout the UV and visible regions, with an estimated optical band gap of approximately 2.76 eV. Li_7_MnN_4_ powder appears black, as shown in the inset of Fig. 3d, consistent with its broad optical absorption across the visible range. This observation supports its potential for light-driven applications in optoelectronics or (photo)catalysis. The UV-vis diffuse reflectance spectrum of Li_7_MnN_4_ shows relatively low *F*(*R*_∞_) values above ∼450 nm. Within the Kubelka–Munk formalism,^32,33^*F*(*R*_∞_) corresponds to the ratio of the absorption coefficient (*K*) to the scattering coefficient (*S*), *i.e.*, *F*(*R*_∞_) = *K*/*S*, and therefore does not directly reflect the intrinsic absorption coefficient. In polycrystalline powder samples with significant light scattering, both the magnitude and wavelength dependence of *S* can substantially influence the apparent spectral intensity. In the visible-NIR region, if electronic transitions are weak while scattering remains pronounced, the resulting *K*/*S* values may appear comparatively small despite the dark macroscopic appearance of the material. Consequently, reduced *F*(*R*_∞_) values at longer wavelengths primarily reflect the interplay of weak transition intensity and scattering rather than an absence of absorption. Comparable behavior has previously been observed in inorganic pigment powders, where pronounced scattering attenuates the apparent Kubelka–Munk response in the visible-NIR range.^34^

The experimental findings were complemented by theoretical calculations performed at the density functional theory (DFT) level. Geometry optimizations of the crystal structure were carried out using the hybrid density functional PW1PW^35^ and the pob-TZVP-rev2 basis sets,^36^ as implemented in the CRYSTAL23 code.^37^ In all calculations, the integral accuracy tolerances (TOLINTEG) were increased to (7, 7, 7, 14, 42), and a 4 × 4 × 4 Monkhorst–Pack *k*-point grid was employed for Brillouin zone sampling. Several magnetic configurations were considered for the eight Mn atoms in the conventional unit cell: diamagnetic (DM), ferromagnetic (FM), ferrimagnetic (FIM), and two antiferromagnetic configurations (AFM1 and AFM2). In the initial electronic configuration of the FIM state, the five *d* electrons of the neutral Mn atoms at the 6*c* sites were assigned spin-up (↑), while those at the 2*a* sites were assigned spin-down (↓). After self-consistent field (SCF) convergence, the resulting atomic spin densities were +2.1 for Mn(6*c*) and −1.7 Mn(2*a*), approximately corresponding to an oxidation state of +5. In the AFM1 configuration, the spin sequence of the eight Mn atoms was set to (↑, ↓, ↑, ↓, ↑, ↓, ↑, ↓), whereas in AFM2 it was (↑, ↓, ↓, ↑, ↑, ↓, ↓, ↑). The resulting atomic spin densities in both AFM configurations were ±1.9. The optimized lattice parameters for all four magnetic states were similar: 9.533 Å (FM), 9.533 Å (FIM), 9.545 Å (AFM1), and 9.532 Å (AFM2); all values are in good agreement with the measured value of 9.560 Å (Table 1). Also, the atomic positions in all four configurations exhibited minimal variation. For reference, the optimized atomic positions of the FM state are given in Table S3. The calculated fractional coordinates of the N atoms closely match the experimental values shown in Table 2, while large deviations are observed for Li4 and Li5. Given the proven accuracy of the PW1PW/pob-TZP-rev2 method for predicting the lattice parameters in similar compounds.^11,13^ these discrepancies may suggest that the experimentally unrefined Li positions obtained from X-ray diffraction analysis are not accurate. The relative energies of the FM, FIM, and AFM states differed by less than 2 kJ mol^−1^, indicating weak coupling between the atomic spins and, consequently, a paramagnetic state, consistent with a previous theoretical study.^24^

**Fig. 4:**
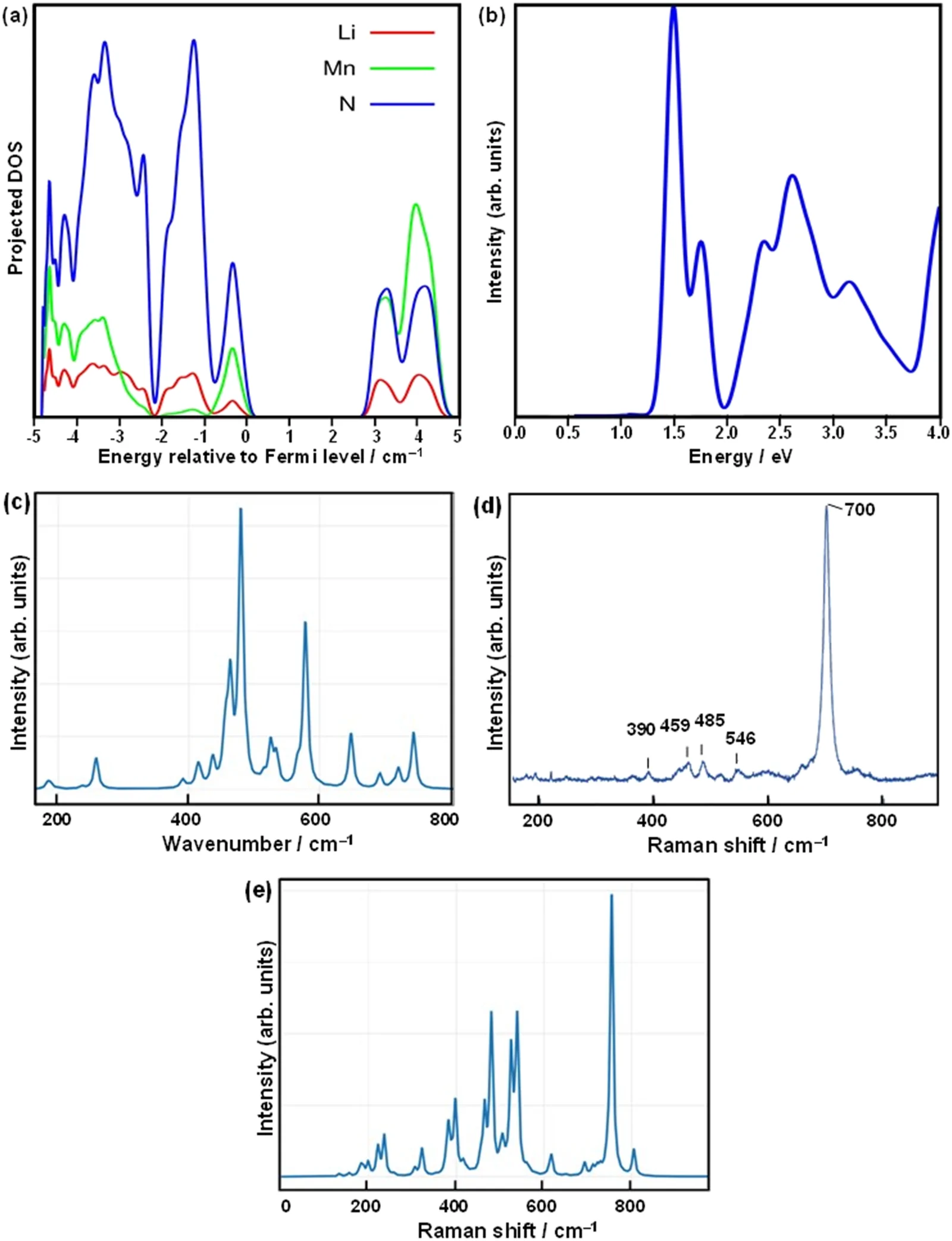
(a) Projected density of states (DOS) of Li_7_MnN_4_ calculated using the sc-PW1PW method with the pob-TZVP-rev2 basis set for the relaxed structure. (b) Optical absorption spectrum of Li_7_MnN_4_ calculated using the GW-BSE method. (c) Infrared spectrum of Li_7_MnN_4_ calculated using the PW1PW method. (d) Experimental Raman spectrum of Li_7_MnN_4_ recorded at 80 K with an excitation wavelength of 488 nm. (e) Raman spectrum of Li_7_MnN_4_ calculated using the PW1PW method. The calculated spectra are plotted using CRYSCOR.

The electronic band gap energy of Li_7_MnN_4_ was calculated using a self-consistent version of the PW1PW hybrid functional, referred to as sc-PW1PW. In sc-PW1PW, the percentage α of Fock exchange in the exchange functional is optimized based on the iteratively calculated dielectric constant *ε*, following the approach described elsewhere.^38^ These calculations revealed a strong dependence of α on the lattice parameters: α was found to be 17.9% for the experimental structure and 20.1% for the optimized structure. The electronic band gap energies calculated with the two variants of sc-PW1PW were 1.45 eV for the experimental structure and 2.96 eV for the optimized structure. Moreover, the nature of the fundamental band gap was found to depend on the structure: a direct band gap at the Y-point (Y → Y) for the experimental structure and an indirect band gap from Z to M (Z → M) for the optimized structure. In the latter case, the direct gap at Γ (Γ → Γ) was only slightly larger (3.05 eV). As in our previous studies,^11,13^ the electronic band gap was also computed at a higher theoretical level for comparison. For these calculations, the GW method, as implemented in VASP (version 6.5.1)^39^ was employed. Due to the large size of the Li_7_MnN_4_ unit cell (96 atoms), a smaller 2 × 2 × 2 Monkhorst–Pack *k*-grid was employed. The plane-wave energy cutoff was set to 500 eV, with 512 bands and 128 frequency points used in the calculation of the response functions. The initial wavefunctions were generated using the PBE functional, and up to six iterations were performed in the self-consistent G_n_W_0_ scheme. For the relaxed structure, a fundamental band gap of 2.74 eV was obtained, slightly smaller than the sc-PW1PW result. Due to the limited Monkhorst–Pack *k*-grid, this band gap corresponds to a Γ → M transition. For the experimental structure, the G_n_W_0_ calculations yielded a direct band gap of 1.76 eV. Although the difference between the band gaps calculated for the experimental and relaxed structures is smaller according to the G_n_W_0_ results than in the sc-PW1PW calculations, the overall trend is consistent: the electronic band gap is larger for the relaxed structure. The projected density of states (DOS) computed using sc-PW1PW for the relaxed structure is shown in Fig. 4a. Both N 2*p* and Mn 3*d* orbitals have significant contributions to the highest occupied and lowest unoccupied bands, whereas the contributions from Li orbitals are relatively small.

The optical absorption spectrum of Li_7_MnN_4_ was computed using the GW-BSE method as implemented in VASP. The quasiparticle band energies were calculated using the G_n_W_0_ approximation described above. In solving the Bethe–Salpeter equation, the 64 highest occupied and 64 lowest unoccupied bands were included. The resulting spectrum is shown in Fig. 4b. In contrast to the experimentally measured optical band gap of 2.76 eV, but consistent with the observed black powder color, the calculated one is approximately 1.0 eV, although the lowest excited states have low intensity. The first intense absorption bands appearing at 1.5–1.7 eV originate from Mn 3d–3d transitions, while the higher-energy features at 2.5–2.7 eV correspond to the N 2p–Mn 3d charge-transfer excitations. Infrared and Raman spectra^40–43^ were computed using the PW1PW method for the relaxed Li_7_MnN_4_ structure in the FIM state (Fig. 4c and e). This configuration exhibits the highest symmetry among the studied magnetic states, thus requiring the least computation effort. The absence of imaginary vibrational frequencies confirms that the relaxed structure corresponds to a local minimum on the potential energy surface. In Fig. 4c, the infrared spectrum exhibits weak absorption at 260 cm^−1^ and intense bands at 460, 480, and 580 cm^−1^. All corresponding vibrational modes have an *F*_2_ symmetry.

The experimental Raman spectrum of Li_7_MnN_4_ powder in Fig. 4d is overall in good agreement with the calculated spectrum in Fig. 4e and closely matches the data reported by Zhou *et al.*^19^ It displays weak signals in the range 200–400 cm^−1^, stronger bands between 400 and 600 cm^−1^, and a distinct, very intense signal between 600 and 800 cm^−1^ (Fig. 4d and e). An overview of all calculated Raman modes is given in Table S4. The spectrum is dominated by modes associated with the MnN_4_ tetrahedra, due to the large changes in polarizability. The band at 390 cm^−1^ can be assigned to Li atom displacements accompanied by deformation vibrations of the MnN_4_ unit. Rotational and deformation modes of the MnN_4_ tetrahedra, with contributions from Li displacements, are observed between 400 and 600 cm^−1^, while the strong and totally symmetric (*A*_1_) breathing vibration of the MnN_4_ tetrahedra at 700 cm^−1^ represents the main feature of the Raman spectrum.^19^ The calculated Raman spectrum exhibits the commonly observed positive offset of the normal mode frequencies,^11^ likely arising from the assumptions and simplifications in the underlying functionals, which do not account for all possible interactions.

The formation energies of Li_7_MnN_4_ were also calculated with PW1PW for two different reaction pathways:4 Li_3_N + Mn → Li_7_MnN_4_ + 5 Li Δ*E* = −122.1 kJ mol^−1^7/3 Li_3_N + 1/4 Mn_4_N + 17/24 N_2_ → Li_7_MnN_4_ Δ*E* = −620.0 kJ mol^−1^These values indicate that the formation of Li_7_MnN_4_ from Li_3_N, Mn_4_N, and N_2_ is energetically more favorable than its formation from Li_3_N and metallic Mn.

The stability of Li_7_MnN_4_ with respect to oxidation was calculated using the PW1PW method. To model the effect of oxidation, one nitrogen atom (N1 or N2) was substituted with an oxygen atom in a supercell, according to the following reaction:Li_56_Mn_8_N_32_ + 1/2 O_2_ → Li_56_Mn_8_N_31_O + 1/2 O_2_The calculations were performed for the FIM state with PW1PW. For both nitrogen substitution sites, the reaction energies were strongly negative (−331 kJ mol^−1^ for N1 and −311 kJ mol^−1^ for N2). These results indicate that Li_7_MnN_4_ is unstable with respect to oxidation. In both cases, the spin density of one Mn atom adjacent to the oxygen atom increased from 2.1 to ∼2.8, suggesting a reduction in the oxidation state of Mn from +5 to +4.

**Fig. 5:**
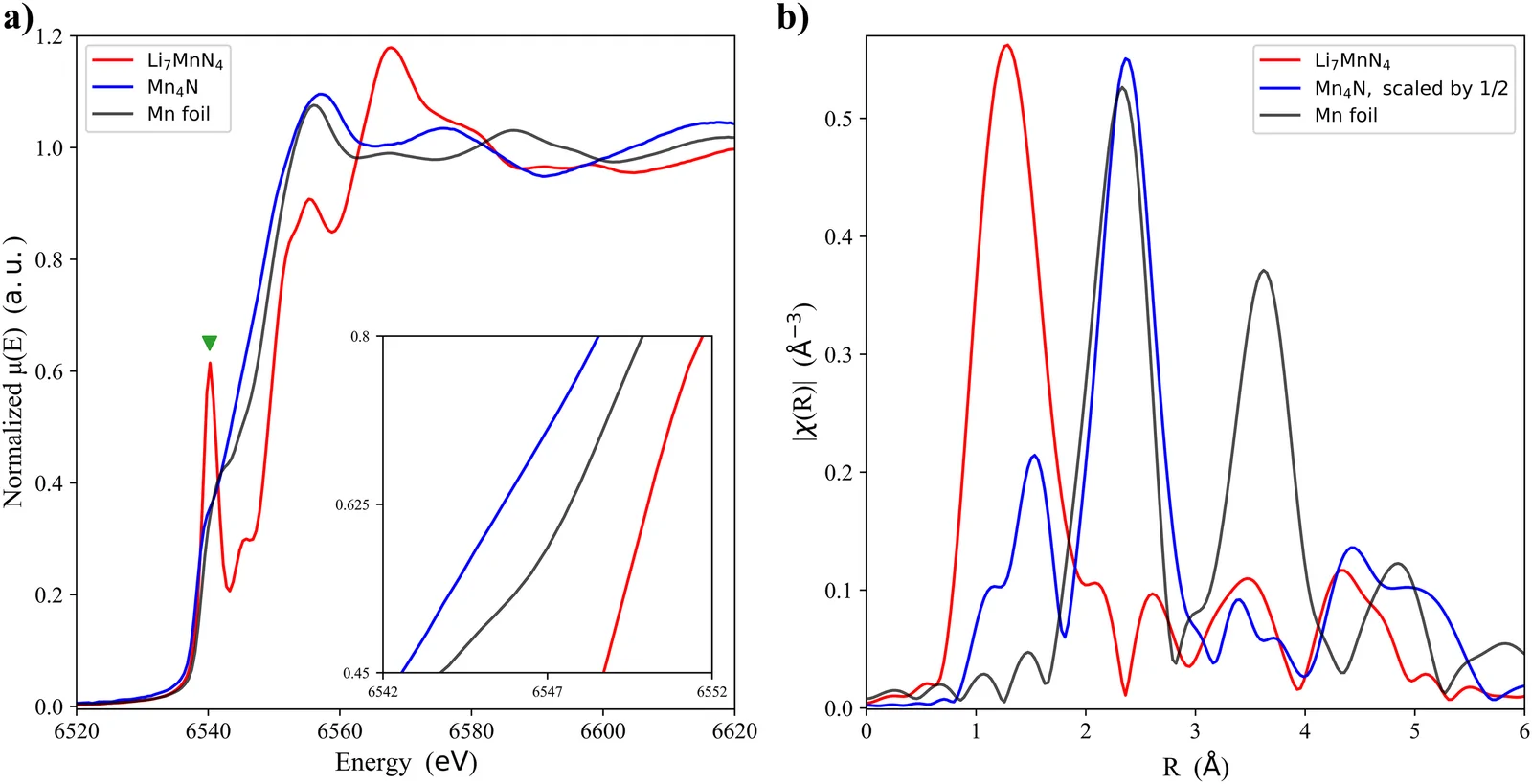
(a) Experimental Mn K-edge XANES of Li_7_MnN_4_ powder. The pre-edge is marked by 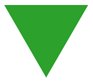, while the inset highlights an enlarged view of the rising edge. (b) Non-phase shift corrected Mn K-edge EXAFS of Li_7_MnN_4_ powder in *R*-space. The spectra are plotted with Mn-based reference materials for comparison.

The local electronic structure around Mn in Li_7_MnN_4_ was probed using X-ray absorption spectroscopy (XAS). The Mn K-edge X-ray absorption near-edge structure (XANES) data of Li_7_MnN_4_ (Fig. 5a) indicates that Mn is in a higher valence state compared to metallic Mn, as evidenced by the shift of the rising edge to higher energies. In addition, the XANES spectrum of Li_7_MnN_4_ exhibits a sharp pre-edge feature (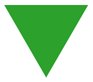) at 6540.3 eV, arising from 1*s* → 3*d* transitions with dipole intensity enabled by 3d–4p orbital mixing.^44,45^ This is a consequence of the non-centrosymmetric Mn–N coordination within the [MnN_4_]^7−^ tetrahedra at both the Mn1 and Mn2 sites. The Mn K-edge extended X-ray absorption fine structure (EXAFS) analysis (Fig. 5b, S3, and S4a, and Table S6) reveals Mn–N and Mn–Li coordination, confirming the highly ordered arrangement of Mn species in Li_7_MnN_4_. The dominant feature in the *R*-space EXAFS spectrum of Li_7_MnN_4_ (Fig. 5b and S4a) primarily arises from Mn–N scattering, while the neighboring shoulder is attributed to Mn–Li contributions. Although Li is a weak backscatterer and is rarely resolved in EXAFS, the Li-rich composition of Li_7_MnN_4_ and the well-separated Mn–Li distances relative to Mn–N render the Mn–Li contributions distinguishable (Fig. S2 and discussion part in SI). Moreover, the interatomic distances extracted from the EXAFS analysis (Table S7) are in good agreement with both the Rietveld refinement presented in this study (*vide supra*) and earlier studies.^15,16^

**Fig. 6:**
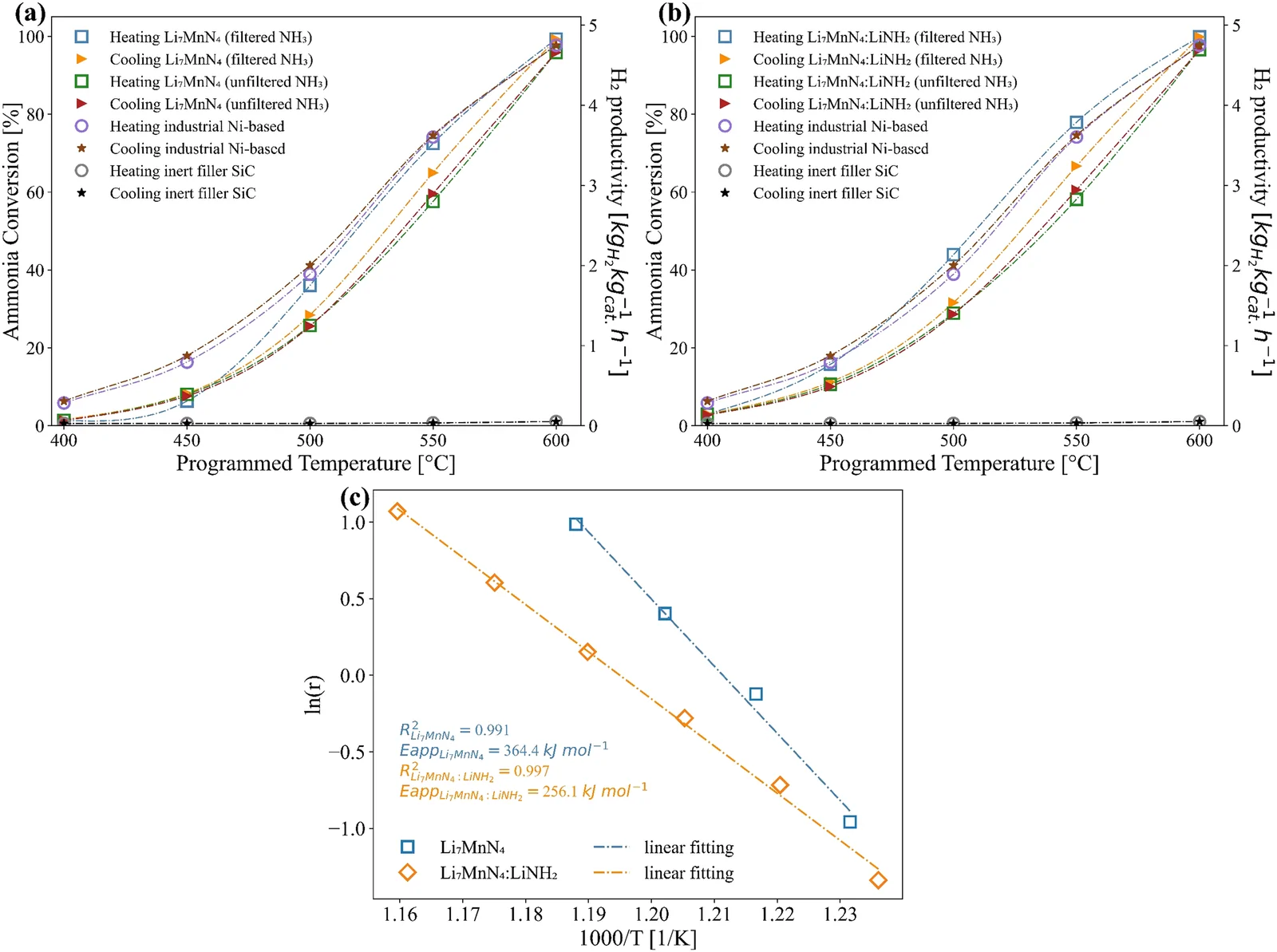
Catalytic ammonia decomposition activities of Li_7_MnN_4_ (a) and Li_7_MnN_4_ : LiNH_2_ (1 : 1 molar ratio) (b) in a temperature range between 400 °C and 600 °C. Reaction conditions: *P* = 1 atm, flow rate = 0.6 mL mg_cat_^−1^ min^−1^, rate of temperature changes between the steps = 2.0 K min^−1^. (c) Arrhenius plots of Li_7_MnN_4_ and Li_7_MnN_4_ : LiNH_2_. Reaction conditions: *P* = 1 atm, flow rate = 4.39 mL mg_cat_^−1^ min^−1^.

The catalytic ammonia decomposition activities of Li_7_MnN_4_ and Li_7_MnN_4_ : LiNH_2_ (1 : 1 molar ratio) are presented in Fig. 6a and b. Both samples exhibit deactivation during the first half of the screening under filtered ammonia flow (see also Fig. S5), as evidenced by the hysteresis observed in the cooling step (*orange curve*). In both cases, the cooling steps coincide with the second screening, indicating that deactivation is significantly reduced. The hysteresis nearly disappears from both samples. This suggests that the presence of trace oxygen in combination with ammonia during the second screening does not adversely affect the catalytic activity. Instead, the apparent deactivation observed during the first screening is likely related to the formation of a new active phase distinct from the original precursors. The new active phase formed during the initial heating–cooling cycle appears to be stable for both compounds, as evidenced by the minimal variability in catalytic activity during subsequent temperature steps. This stability is reflected in the near overlap of the mean ammonia conversion values in Fig. 6a and b at different temperature steps. In addition, the time-on-stream data presented in Fig. S5 further confirm the stability of this active phase, showing consistent conversion levels at the same temperatures as the reaction proceeds. Throughout the entire experiment, Li_7_MnN_4_ : LiNH_2_ (1 : 1 molar ratio) consistently shows higher activity for catalytic ammonia decomposition compared to Li_7_MnN_4_ alone. Moreover, in the first segment of the experiment, at temperature steps above 450 °C, its catalytic activity even surpasses that of the Ni-based reference catalyst. The apparent activation energies of the two samples differ markedly: 364.4 kJ mol^−1^ for Li_7_MnN_4_ and 256.0 kJ mol^−1^ for Li_7_MnN_4_ : LiNH_2_ (1 : 1 molar ratio) (Fig. 6c). This substantial difference indicates that the active phases formed from the two precursors are distinct, with the new phase derived from Li_7_MnN_4_ : LiNH_2_ (1 : 1 molar ratio) being intrinsically more active toward ammonia decomposition. The incorporation of LiNH_2_ appears to have a beneficial effect on the catalytic behavior of Li_7_MnN_4_ under the applied reaction conditions, leading to enhanced overall activity.

To the best of our knowledge, Li_7_MnN_4_ has not previously been studied as a catalyst for ammonia decomposition. A previous study by Chang *et al.*^46^ using a 1 : 1 MnN : LiNH_2_ composite reported an apparent activation energy of 79.8 kJ mol^−1^, with comparable ammonia conversions under similar reaction conditions (flow rate of 0.6 mL mg_cat_^−1^ min^−1^ in the present work *vs.* 0.667 mL mg_cat_^−1^ min^−1^ in Chang *et al.*^46^). The significantly higher activation energies observed in this study suggest the formation of different active phases in both Li_7_MnN_4_ and Li_7_MnN_4_ : LiNH_2_, likely due to variations in the Li : Mn ratio between the two precursors. On the contrary, another study^8^ investigated MnN : LiNH_2_ composites across a wide range of ratios (1 : 0.2 to 1 : 23), which included the ratio relevant here, and reported only minor variations in apparent activation energy, ranging from 72.2 kJ mol^−1^ and 78.5 kJ mol^−1^.^8^

Their findings lead to two possible but contrasting interpretations: either similar active phases can form across different Li : Mn ratios, or the nature of the precursor governs the formation of the active phase rather than the ratio itself. Guo *et al.*^8^ further proposed a reaction pathway *via* a redox cycle with Li_7_MnN_4_ as an intermediate. In this reaction pathway, Li_7_MnN_4_ reacts with ammonia to generate MnN, Li_2_NH, and N_2_, completing the catalytic cycle. However, it was also suggested that LiNH_2_, produced from the reaction between Li_2_NH and ammonia, may melt and volatilize under a high ammonia partial pressure, leading to lithium loss from the system. This appears to be the case in the present study. Such lithium depletion could explain the deactivation observed during the first half of the initial screening, as it results in the loss of lithium-containing active species.

It should be noted that the apparent activation energy values reported in the literature were not obtained under conditions close to kinetic control, unlike in this study. Here, ammonia was diluted to 50% in helium to reduce the impact of the endothermicity of the reaction on the temperature profile of the catalytic bed, and the use of reaction rate values from temperature steps, where the conversion was less than 10%, was applied to reduce the impact of the concentration profiles that can be created in the catalytic bed. The apparent activation energy values obtained without taking these criteria into account lead to results that do not reflect intrinsic kinetics.

**Fig. 7:**
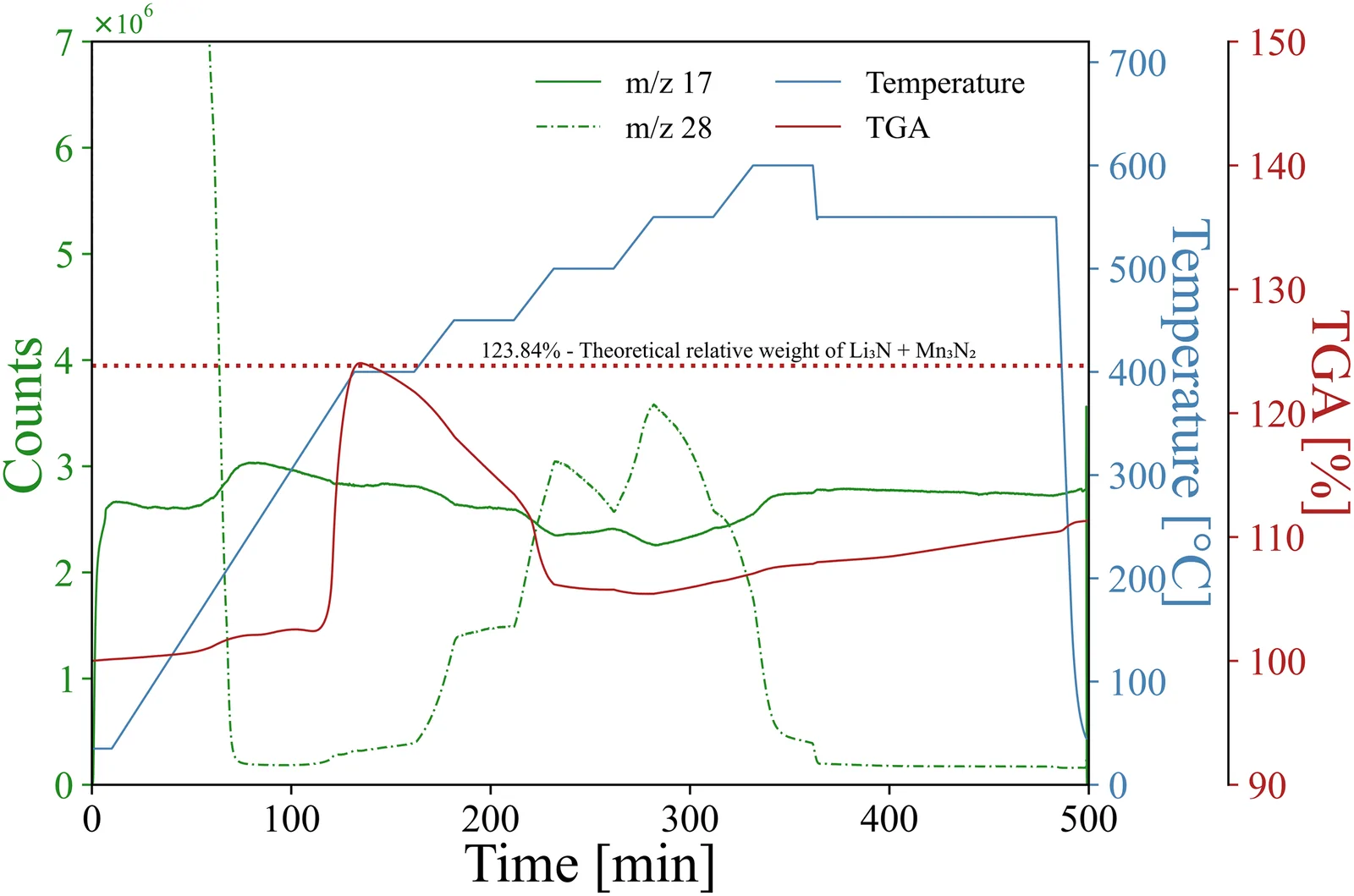
TG-MS results of Li_7_MnN_4_ obtained under an NH_3_ flow.

A thermogravimetry-mass spectrometry (TG-MS) analysis performed under an NH_3_ flow provided deeper insights into the changes occurring in Li_7_MnN_4_ during the reaction. Fig. 7 shows the weight changes induced by the temperature steps, which mimic the activity test program, together with the evolution of signals at masses 17 and 28. The weight change reached a maximum of 124.04%, while at the end of the experiment, it stabilized at 111.29% during the cooling. The activity, as monitored by the mass variations, revealed weak conversion at 400 °C, a clearly detectable and time-stable ammonia decomposition at 450 °C, and a pronounced deactivation above 500 °C, with practically no activity remaining at 600 °C. The combined data indicate that conversion starts at 355 °C, coinciding with the onset of the weight increase. However, when the weight increase reaches its inflection point at 400 °C, the signal at mass 28 continues to rise monotonically without any apparent change. This suggests that the weight increase reflects modifications in the solid matrix that are not directly related to the active phase.

The maximum weight increase of 24.04% is very close to the theoretical value of 23.84%, which corresponds to the formation of Li_3_N and Mn_3_N_2_ if all the metals in Li_7_MnN_4_ were to react and form these binary nitrides. All other possible combinations of compounds, including those involving the formation of LiNH_2_ or Li_2_NH together with any Mn-based phase, yield theoretical weight values significantly higher than the experimental result. A previous study reported that LiNH_2_ decomposes above 372 °C to form Li_2_NH and NH_3_.^47^ Further decomposition may ultimately produce Li_3_N, following the reverse pathway of the hydrogenation reactions described previously in studies of H_2_ storage.^48^ Therefore, the decomposition of Li_7_MnN_4_ may proceed initially through the formation of amide and imide intermediates before yielding the binary nitrides. As mentioned earlier, both LiNH_2_ and Li_2_NH can melt under the reaction conditions, which would lead to the coexistence of at least two phases: a Li-enriched melt and an Mn-enriched solid. Unlike in the tubular reactor, where molten solids can seep into the interparticle voids of the catalyst and SiC bed until solidifying in a cooler section of the quartz tube, the crucible used in the TG-MS analysis acts as a container that prevents the melt from permeating. As a result, Mn-based species become covered by Li compounds. Evidence of this melting process was provided by the observation that the sample, initially in powder form, developed into a rigid structure by the end of the test (Fig. S6, SI).

In any case, the formation of new phases by the incorporation of N and/or H into Li and Mn ceases at 400 °C. This process, like the initial weight increase, does not affect the conversion: the signal at mass 28 continues to increase monotonically during the 450 °C step, following the sharp rise observed during heating from 400 °C. It is only upon heating to 500 °C that both parameters change simultaneously, as evidenced by a sharp drop in weight and activity starting at 475 °C. At 500 °C, the weight stabilizes again, but the activity continues to decrease. The more stable solid phase formed at the surface is most likely Li-rich and practically inactive above 550 °C. The minimum weight recorded between 500 °C and 550 °C was 105.4%, which is close to the theoretical value expected for a mixture of LiH and Mn_6_N_5−6_ (Table S8, SI). The subsequent progressive weight increase, which continued until the end of the test, approaches the theoretical value for Li_3_MnN_2_ and metallic Li. However, the presence of metallic Li is improbable. A more likely explanation is that the final composition consists of LiH and Li_3_MnN_2_, which corresponds to a theoretical relative weight of 115.35%. The formation of Li_3_MnN_2_ under these conditions may be slow and incomplete, since the reaction occurs within the solid matrix where Mn species are covered by Li compounds, hindering the access of N to Mn. The degree of activity loss observed in the TG-MS result was not detected during the regular catalytic activity tests. This discrepancy stems from the different reactor geometries: in the tubular reactor, Li imides and amides can migrate out of the catalyst bed, leaving behind a Mn-rich phase, which appears to be more catalytically active than any Li-containing compound formed.

In general, both Li_7_MnN_4_ and Li_7_MnN_4_ : LiNH_2_ (1 : 1 molar ratio) exhibit catalytic ammonia decomposition activities comparable to that of the reference Ni-based catalyst. However, due to the deactivation observed during the first half of the initial screening, the Ni-based catalyst ultimately remains more active. Nevertheless, the presented findings demonstrate the high potential of lithium-manganese nitrides as active catalysts for ammonia decomposition. Future studies should explore the effects of promoters, supports, modified pretreatment conditions, or their combinations to further improve the catalytic activity and stability of these materials.

## 4. Conclusions

In this work, antifluorite-derived Li_7_MnN_4_ was synthesized in phase-pure powder form by a simplified solid-state reaction, and its cubic structure, crystallizing in space group *P*4̄_3_*n* (no. 218) and featuring isolated [MnN_4_]^7−^ tetrahedra and five distinct Li sites, was refined by Rietveld refinement. Density functional theory calculations revealed weak spin coupling, consistent with a paramagnetic ground state. Complementary spectroscopic (UV-vis, Raman, and XAS) investigations confirmed its narrow optical band gap, dominant Mn–N vibrations, and a high Mn oxidation state in a well-defined Mn–N/Li coordination environment. Both Li_7_MnN_4_ and Li_7_MnN_4_ : LiNH_2_ (1 : 1 molar ratio) exhibited catalytic activities for ammonia decomposition, which is comparable to a Ni-based reference catalyst. Particularly, the incorporation of LiNH_2_ significantly reduced the apparent activation energy, highlighting its beneficial role in generating more active phases under reaction conditions. The catalytic ammonia decomposition proceeded *via* the formation of LiNH_2_/Li_2_NH intermediates, as revealed by TG-MS. These results demonstrate that the synergy between lithium amide-imide chemistry and Mn–N bonding is essential for N-H bond activation. Even with the known sensitivity of nitride-based compounds to exposure to air, the industry already works with nitrides as catalysts that always require handling in an inert atmosphere. The established strategies to address this challenge include *in situ* synthesis, encapsulation or transport in airtight containers, and the use of airlocks. Li_7_MnN_4_ enriches the emerging family of lithium transition metal nitrides as promising catalysts for ammonia decomposition.

## Author contributions

Mirabbos Hojamberdiev: conceptualization, formal analysis, investigation, methodology, visualization, writing – original draft, writing – review & editing; Ana Laura Larralde: formal analysis, software, validation, visualization, writing – original draft, writing – review & editing; Eva M. Heppke: formal analysis, software, validation, visualization, writing – original draft, writing – review & editing; Oscar Gómez-Cápiro: formal analysis, investigation, methodology, validation, visualization, writing – original draft, writing – review & editing; John Carl A. Camayang: formal analysis, investigation, methodology, software, validation, visualization, writing – original draft, writing – review & editing, Thomas Bredow: investigation, methodology, software, validation, visualization, writing – original draft, writing – review & editing; Kunio Yubuta: investigation, methodology, visualization, writing – original draft, writing – review & editing; Katsuya Teshima: investigation, methodology, visualization, writing – original draft, writing – review & editing; Tamanna M. Ahamad: investigation, methodology, visualization, writing – original draft, writing – review & editing; Christian Lorent: investigation, methodology, visualization, writing – original draft, writing – review & editing; Liqun Kang: formal analysis, investigation, methodology, visualization, writing – original draft, writing – review & editing; Yves Kayser: formal analysis, investigation, methodology, visualization, writing – original draft, writing – review & editing; Holger Ruland: funding acquisition, investigation, project administration, resources, supervision, writing – original draft, writing – review & editing; Serena DeBeer: funding acquisition, investigation, project administration, resources, supervision, writing – original draft, writing – review & editing; Martin Lerch: funding acquisition, investigation, project administration, resources, supervision, writing – original draft, writing – review & editing.

## Conflicts of interest

There are no conflicts of interest to declare.

## Supplementary Material

CY-016-D5CY01547B-s001

## Data Availability

The XAFS datasets, both raw and processed, have been uploaded to the EDMOND repository and can be accessed using the following link: https://doi.org/10.17617/3.2O31P5. The rest of the data that support the findings of this study are available from the corresponding author upon request. Supplementary information (SI) is available. See DOI: https://doi.org/10.1039/d5cy01547b.
