# Supplementary material for: Antifluorite-derived Li7MnN4: revisiting the crystal structure and catalysis in ammonia decomposition
Source: Catal Sci Technol. 2026 Mar 9;16(8):2836–49. doi: 10.1039/d5cy01547b (PMC12990304; doi:10.1039/d5cy01547b)
Supplement: CY-016-D5CY01547B-s001 [file CY-016-D5CY01547B-s001.pdf]

Supplementary Information (SI) for Catalysis Science & Technology.
This journal is © The Royal Society of Chemistry 2026

Electronic Supplementary Information (ESI)

Antifluorite-Derived Li_7MnN_4: Revisiting the Crystal Structure and Catalysis in Ammonia Decomposition

Mirabbos Hojamberdiev[1,2*], Ana Laura Larralde[3,4], Eva M. Heppke[1], Oscar Gómez-Cápiro[5], John Carl A. Camayang[6], Thomas Bredow[7], Kunio Yubuta[8], Katsuya Teshima[8], Tamanna M. Ahamad[1], Christian Lorent[1], Liqun Kang[6], Yves Kayser[6], Holger Ruland[5], Serena DeBeer[6], and Martin Lerch[1]

[1]Institut für Chemie, Technische Universität Berlin, Straße des 17. Juni 135, 10623 Berlin, Germany

[2]Mads Clausen Institute, University of Southern Denmark, Alsion 2, 6400 Sønderborg, Denmark

[3]Consejo Nacional de Investigaciones Científicas y Técnicas (CONICET), Buenos Aires, Argentina

[4]Instituto Nacional de Tecnología Industrial, Avenida General Paz 5445, San Martín (B1650WAB), Buenos Aires, Argentina

[5]Department of Heterogeneous Reactions, Max Planck Institute for Chemical Energy Conversion, Stiftstraße 34–36, 45470 Mülheim an der Ruhr, Germany

[6]Department of Inorganic Spectroscopy, Max Planck Institute for Chemical Energy Conversion, Stiftstraße 34–36, 45470 Mülheim an der Ruhr, Germany

[7]Mulliken Center for Theoretical Chemistry, Clausius-Institut für Physikalische und Theoretische Chemie, University of Bonn, Beringstraße 4, 53115 Bonn, Germany

[8]Institute for Aqua Regeneration, Shinshu University, 4-17-1 Wakasato, Nagano 380-8553, Japan

*Corresponding author: E-mail addresses: mirabbos@mci.sdu.dk and hmirabbos@gmail.com (M. Hojamberdiev)

1. Supplementary Methods: X-ray absorption measurements

The Mn K-edge XAFS spectra of Li_7MnN_4 powder were collected at BM31 of SNBL at the ESRF [1–4]. During the measurements, the ESRF's Extremely Brilliant Source (ESRF-EBS) was operated at 6 GeV with a 200 mA ring current in top-up mode, using the 7/8 + 1 hybrid filling pattern [5]. At BM31, the X-ray beam is generated from the 0.86 T section of a standard ESRF 2-Pole Wiggler (2PW), which matches the multi-bend achromat lattice of the ESRF-EBS. A cooled aperture plate with slits splits the X-ray beam into two parts: BM31 receives 1 mrad of the upstream beam, separated by 2.5 mrad from SNBL's BM01 branch, which operated in parallel. The Mn K-edge measurements were performed using an air-bearing, liquid-N_2-cooled Si(111) double-crystal monochromator (DCM), consisting of two pairs of flat crystals. The DCM provided a resolving power of $\Delta E/E = 10^{-4}$, with a maximum photon flux of 10^{10} ph/s and a beam spot size of 0.6 mm × 1 mm (V × H) [3,4]. The incident photon energy of the DCM was calibrated using a 5 μm Mn foil, where the energy of the first inflection point in the first derivative of the Mn foil XANES spectrum was set to 6539 eV. XAFS data for both the sample and the reference foil were collected simultaneously in transmission mode using ionization chambers, with the reference foil positioned downstream of the sample. The XAFS spectra of the samples were then aligned to the corresponding Mn foil XANES spectra. Full XAFS spectra at the Mn K-edge were recorded in the range 6440-7140 eV, with a fine step size of 0.45 eV across the rising edge region (6520-6570 eV). In addition to Li_7MnN_4, Mn_4N was also measured as a reference for subsequent data analysis. As described in the main text's Methods section, Li_7MnN_4 and Mn_4N powders were mixed and homogenized with a BN binder in composition-dependent mass ratios to optimize transmission properties. The sample-to-BN mass ratios were 1:9 for Li_7MnN_4 and 1:19 for Mn_4N.

The XAFS spectra were analyzed using the xraylarch software package (version 0.9.74) [6]. Pre-edge background subtraction and post-edge normalization of the XAFS data were performed using the same program, with E_0 defined as the energy of the first inflection point in the first derivative XANES for both Mn_4N and Mn foil. However, for Li_7MnN_4, this inflection point coincides with the pre-edge feature. Therefore, E_0 was set to the energy of the second inflection point. Linear regression was applied to determine the pre-edge background, while a quadratic polynomial was used for post-edge normalization. Unless otherwise specified (*vide infra*), a spline function within the range k = 0-12.5 Å^{-1} and R_{bkg} = 1 Å, together with a Kaiser-Bessel window (dk = 1), was employed to generate the forward Fourier-transformed EXAFS data. The fitting of Mn K-edge EXAFS spectra was performed in xraylarch using scattering paths generated by FEFF8L. Wavelet transform EXAFS (WT-EXAFS) spectra were computed using the Cauchy wavelet transform function implemented in the xraylarch Python library (version 0.9.74) [6,7].

2. Supplementary Tables

Table S1. Comparison of bond lengths (Å) for Li_7MnN_4.

Bonds	Powder neutron diffraction data [8]	Single crystal X-ray diffraction data [9]	Powder X-ray diffraction data *(this study)*
Mn2 – N2	4 × 1.808(6)	4 × 1.810(4)	4 × 1.787(7)
Mn1 – N1	4 × 1.852(4)	4 × 1.826(3)	4 × 1.868(4)
Li5 – N2	2 × 2.06(4)	2 × 2.086(5)	2 × 2.087(7)
Li5 – N1	2 × 2.16(3)	2 × 2.164(5)	2 × 2.122(5)
Li2 – N1	4 × 2.121(4)	4 × 2.122(2)	4 × 2.114(5)
Li1 – N1	4 × 2.045(4)	4 × 2.037(2)	4 × 2.036(4)
Li3 – N1	3 × 2.21(3)	3 × 2.220(3)	3 × 2.198(4)
Li3 – N2	1 × 2.05(3)	1 × 2.050(8)	1 × 2.121(7)
Li4 – N1	1 × 2.05(3)	1 × 2.042(6)	1 × 2.036(4)
Li4 – N1	1 × 2.08(3)	1 × 2.096(5)	1 × 2.119(4)
Li4 – N1	1 × 2.14(3)	1 × 2.154(6)	1 × 2.119(4)
Li4 – N2	1 × 2.18(3)	1 × 2.192(6)	1 × 2.198(7)

Table S2. Comparison of bond angles (°) for Li_7MnN_4.

Bonds	Powder neutron diffraction data [8]	Single crystal X-ray diffraction data [9]	Powder X-ray diffraction data *(this study)*
N2–Mn2–N2	6 × 109.5(5)	6 × 109.47	6 × 109.5(6)
N1–Mn1–N1	4 × 108.3(3)	4 × 108.35(6)	4 × 108.7(4)
	2 × 111.9(2)	2 × 111.7(1)	2 × 111.0(3)
N1–Li1–N1	2 × 97.3(3)	2 × 95.79(15)	2 × 98.3(3)
	2 × 113.1(2)	2 × 113.21(12)	2 × 112.8(2)
	2 × 118.8(2)	2 × 120.33(10)	2 × 118.0(4)
N1–Li2–N1	2 × 107.1(3)	2 × 106.54(9)	2 × 106.6(4)
	4 × 110.7(3)	4 × 110.96(4)	4 × 110.9(3)
N1–Li3–N1	3 × 116.7(11)	3 × 107.52(18)	3 × 107.6(3)
N1–Li3–N2	3 × 112.1(12)	3 × 111.36(17)	3 × 111.3(4)
N1–Li4–N1	1 × 93.3(12)	1 × 91.3(3)	1 × 93.9(3)
	1 × 111.6(11)	1 × 110.7(2)	1 × 110.5(4)
	1 × 115.2(11)	1 × 117.2(3)	1 × 117.2 (2)
N1–Li4–N2	1 × 110.3(12)	1 × 110.7(2)	1 × 110.0(4)
	1 × 112.0(11)	1 × 111.2(2)	1 × 111.3(4)
	1 × 113.0(11)	1 × 113.6(2)	1 × 112.5(4)
N1–Li5–N1	1 × 109.1(12)	1 × 109.5(3)	1 × 110.6(4)
N1–Li5–N2	2 × 110.6(13)	2 × 110.54(9)	2 × 111.1(4)
	2 × 117.2(13)	2 × 117.65(8)	2 × 117.0(4)
N2–Li5–N2	1 × 91.4(15)	1 × 90.2(4)	1 × 88.7(4)

Table S3. Calculated atomic parameters for Li_7MnN_4 (PW1PW results for the FM state). The deviations in parentheses are from the values obtained from Rietveld refinement in Table 2.

Atom	Wyckoff	*x*	*y*	*z*
Li1	6*b*	0	1/2	1/2
Li2	6*d*	1/4	0	1/2
Li3	8*e*	0.2322(0.0082)	0.2322(0.0082)	0.2322(0.0082)
Li4	24*i*	0.2499(0.0160)	0.2385(0.0111)	-0.0179(-0.0099)
Li5	12*f*	0.2639(0.0299)	0	0
Mn1	6*c*	1/2	0	1/4
Mn2	2*a*	0	0	0
N1	24*i*	0.3560(-0.0043)	0.3839(0.0021)	0.1062(-0.0028)
N2	8*e*	0.1089(0.0003)	0.1089(0.0003)	0.1089(0.0003)

Table S4. Normal modes with respective frequencies (cm^{-1}) and Mulliken symbols as well as the corresponding single crystal directional intensities (arbitrary units), calculated using the PW1PW method.

MODES		FREQUENCIES		*I*_xx	*I*_xy	*I*_xz	*I*_yy	*I*_yz	*I*_zz
4-	6	133.5738	F_2	0	2.22	2.22	0	2.22	0
10-	12	155.4520	F_2	0	2.60	2.60	0	2.60	0
16-	17	179.9734	E	14.49	0	0	14.49	0	14.46
18-	20	183.5886	F_2	0	5.13	5.13	0	5.13	0
21-	23	188.1464	F_2	0	5.41	5.41	0	5.41	0
27-	29	199.2475	F_2	0	10.69	10.68	0	10.68	0
36-	38	221.4259	F_2	0	24.57	24.58	0	24.57	0
39-	41	235.5584	F_2	0	34.22	34.22	0	34.22	0
45-	45	251.0454	A_1	1.17	0	0	1.17	0	1.17
46-	48	257.6964	F_2	0	0.67	0.67	0	0.67	0
49-	50	258.0372	E	3.66	0	0	3.66	0	3.65
58-	60	305.3506	F_2	0	6.84	6.84	0	6.84	0
61-	63	321.5578	F_2	0	23.45	23.44	0	23.44	0
64-	65	322.5554	E	0.56	0	0	0.56	0	0.56
72-	73	354.2121	E	3.35	0	0	3.34	0	3.35
84-	85	376.0306	E	19.66	0	0	19.66	0	19.66
86-	88	382.3269	F_2	0	39.38	39.38	0	39.38	0
89-	91	389.7576	F_2	0	9.57	9.56	0	9.56	0
95-	97	390.4090	F_2	0	0.29	0.29	0	0.29	0
101-	102	396.2248	E	1.35	0	0	1.35	0	1.35
103-	105	398.1978	F_2	0	59.67	59.67	0	59.66	0
109-	109	404.6644	A_1	0.01	0	0	0.01	0	0.01
111-	113	405.6736	F_2	0	0	0	0	0	0
114-	115	410.6754	E	3.05	0	0	3.05	0	3.05
119-	121	413.4779	F_2	0	1.72	1.72	0	1.72	0
122-	124	416.2686	F_2	0	7.47	7.46	0	7.47	0
125-	127	418.0921	F_2	0	0.10	0.10	0	0.10	0
131-	132	423.9460	E	10.53	0	0	10.53	0	10.52
137-	137	431.1748	A_1	0.51	0	0	0.51	0	0.51
138-	139	431.6098	E	0.80	0	0	0.80	0	0.80
141-	143	436.4424	F_2	0	2.20	2.20	0	2.20	0
147-	149	454.2238	F_2	0	4.03	4.02	0	4.02	0
150-	151	455.3265	E	24.31	0	0	24.32	0	24.30
152-	154	456.8842	F_2	0	1.06	1.06	0	1.06	0
158-	160	463.3648	F_2	0	3.14	3.14	0	3.14	0
161-	162	464.7294	E	148.55	0	0	148.55	0	148.6
164-	164	474.4483	A_1	4.45	0	0	4.45	0	4.45
165-	166	479.3918	E	184.94	0	0	184.94	0	184.98
167-	169	479.5009	F_2	0	46.99	47.01	0	47.01	0
176-	177	481.8713	E	92.26	0	0	92.25	0	92.22
178-	180	487.0417	F_2	0	0.35	0.35	0	0.35	0
184-	185	499.0907	E	21.05	0	0	21.05	0	21.05
186-	188	504.7490	F_2	0	9.30	9.30	0	9.30	0
189-	189	505.5321	A_1	29.18	0	0	29.18	0	29.17
190-	191	506.4459	E	23.44	0	0	23.43	0	23.45

195-	197	514.1864	F_2	0	4.08	4.08	0	4.08	0
202-	204	525.0853	F_2	0	99.38	99.40	0	99.40	0
206-	207	533.6015	E	50.69	0	0	50.70	0	50.66
208-	210	533.6430	F_2	0	0.11	0.11	0	0.11	0
214-	216	538.9804	F_2	0	122.92	122.90	0	122.90	0
220-	221	544.8020	E	5.72	0	0	5.72	0	5.72
222-	222	560.2499	A_1	18.91	0	0	18.90	0	18.90
223-	225	566.3007	F_2	0	3.12	3.12	0	3.12	0
229-	231	578.4106	F_2	0	0.81	0.81	0	0.81	0
235-	237	600.5590	F_2	0	0.43	0.43	0	0.43	0
239-	239	609.5735	A_1	0.84	0	0	0.84	0	0.84
240-	241	611.5701	E	7.69	0	0	7.69	0	7.69
245-	247	616.6845	F_2	0	17.33	17.33	0	17.33	0
251-	253	648.3758	F_2	0	1.64	1.63	0	1.63	0
257-	259	692.4443	F_2	0	11.05	11.05	0	11.05	0
266-	268	711.2165	F_2	0	6.28	6.28	0	6.28	0
270-	272	720.6686	F_2	0	5.92	5.92	0	5.92	0
273-	274	728.2893	E	16.18	0	0	16.18	0	16.17
275-	277	744.1045	F_2	0	6.31	6.31	0	6.31	0
278-	278	754.4877	A_1	1000	0	0	999.95	0	999.89
282-	283	773.9958	E	0.75	0	0	0.75	0	0.75
287-	287	804.8427	A_1	92.26	0	0	92.26	0	92.29

Table S5. Structural parameters obtained from EXAFS fitting of the Mn K-edge data of Mn_4N. The fitting considered the first (Mn–N) and second (Mn–Mn) coordination shells. The interatomic distance (R), atomic coordination number (CN), and Debye-Waller factor (σ^2) were extracted using the xraylarch program [6]. The ionization threshold energy (E_0), which propagates the photoelectron's kinetic energy in the calculation, was also determined during the fit. The R-factor and reduced χ^2 are included as statistical parameters that describe the goodness of the fit. The Mn K-edge XAFS data of Mn_4N were collected *ex situ* at BM31/SNBL at the ESRF (see details in the Materials Characterization section).

Scattering Path	CN (fixed)	R (Å)	σ^2 (Å^2)	E_0 (eV)	R-factor	Reduced χ^2
Mn1–Mn2	12	2.752(12)	0.0076(11)	6539.86 ± 1.42	0.0256	150.94
Mn2–N	2	1.95(9)	0.002(4)			
Mn2–Mn1	4	2.752(12)	0.0076(11)			

The Kaiser-Bessel window (dk = 1) was applied during the forward Fourier transform to obtain the R-space data for fitting. The fitting employed a k-range of 2-13 Å^{-1} and an R-range of 1.1-3 Å (see fitting window in Figure S1b), yielding 14.31 independent points compared to the four refined parameters (R, σ^2, S_0^2, and ΔE_0). The primary objective of the EXAFS fitting of the Mn_4N data was to determine the amplitude reduction factor (S_0^2), an element-specific parameter subsequently fixed in the EXAFS fitting of the Mn K-edge data of Li_7MnN_4. In this case, S_0^2 was found to be 0.592.

Table S6. Structural parameters obtained from EXAFS fitting of the Mn K-edge data of Li_7MnN_4. The fitting considered five scattering paths, with interatomic distances (R) and Debye-Waller factor (σ^2) extracted. The R-factor and reduced χ^2 are included as statistical parameters that describe the goodness of the fit. The Mn K-edge XAFS data of Li_7MnN_4 were collected *ex situ* at BM31/SNBL at the ESRF (see details in the Materials Characterization section).

Absorber	Scattering Path	CN	σ^2 (Å^2) *	E_0 (eV)	R-factor	Reduced χ^2
Mn1	Mn1–N1	3.74 ± 0.48	0.0065	6545.53 ± 2.76	0.0615	53.30
	Mn1–Li1	2.26 ± 0.29	0.0053			
	Mn1–Li4	4.52 ± 0.58	0.0054			
Mn2	Mn2–N2	3.74 ± 0.48	0.0065			
	Mn2–Li5	6.78 ± 0.87	0.0052			

*To avoid over-parameterization, the Debye-Waller factors (σ^2) for each scattering path were pre-determined. As shown in the EXAFS equation (see equation S.1), σ^2 and coordination number (CN) are strongly anticorrelated parameters. Hence, allowing all σ^2 and N parameters to vary freely can lead to unstable fits and non-physical uncertainties. The Debye-Waller factors were obtained from prior calculations using the *sigma2_debye* function, which is included in the *sigma2_models* module of the xraylarch Python library [6]. This approach applies the Correlated Debye (CD) model, which requires the material's Debye temperature as input. Since no experimental value for Li_7MnN_4 is currently available, the Debye temperature of a related Mn-based nitride [10] was adopted for the calculations. This approximation is considered reasonable for the present analysis, as the primary objective herein is the comparative evaluation of coordination numbers and bond distances relative to crystallographic information from diffraction studies.

The Kaiser-Bessel window (dk = 1) was applied during the forward Fourier transform to obtain the R-space data for fitting. The fitting employed a k-range of 2.5-12.5 Å^{-1} and an R-range of 1-2.5 Å (fitting window shown in Figures S2b-d), yielding 10.31 independent points compared to five refined parameters (ΔE_0, CN_{1st_shell}, CN_{2nd_shell}, r_{1st_shell}, and r_{2nd_shell}). The r parameters were introduced as contraction/expansion factors, applied multiplicatively to the interatomic distances (R) of the five scattering paths considered. Since all paths describe scattering vectors within a single crystalline lattice, the corresponding contraction/expansion deviations are inherently interrelated.

Table S7. Comparison of selected interatomic distances (Å) for Li_7MnN_4, involving Mn.

Bonds	Powder neutron diffraction data [8]	Single crystal X-ray diffraction data [9]	Powder X-ray diffraction data *(this study)*	EXAFS *(this study)*
Mn1–N1	4 × 1.852(4)	4 × 1.826(3)	4 × 1.868(4)	4 × 1.812(4)
Mn1–Li1	2 × 2.389(8)	2 × 2.39(3)	2 × 2.389(5)	2 × 2.384(5)
Mn1–Li4	4 × 2.53(3)	4 × 2.514(6)	4 × 2.506(4)	4 × 2.521(5)
Mn2–N2	4 × 1.808(6)	4 × 1.81(4)	4 × 1.787(7)	4 × 1.772(4)
Mn2–Li5	6 × 2.46(5)	6 × 2.517(7)	6 × 2.523(7)	6 × 2.451(5)

Table S8. Theoretical weight percentages of the phases formed from the decomposition of Li_7MnN_4, calculated under the assumption of complete conversion of Mn and Li into the corresponding products.

	Li	LiH	$LiNH_2$	Li_2NH	Li_3N
Mn	88.07	73.79	183.53	132.8	115.89
MnN	100	106.00	195.45	144.72	127.81
Mn_2N	94.04	79.76	189.49	138.76	121.85
Mn_3N_2	96.03	102.03	191.48	140.75	123.84
Mn_4N	91.06	97.06	186.51	135.78	118.87
Mn_6N_5	98.01	104.02	193.46	142.74	125.83
Mn_6N_6	100	106.00	195.45	144.72	127.81
Li_3MnN_2	111.92	115.35	166.46	137.48	127.81

3. Supplementary Figures

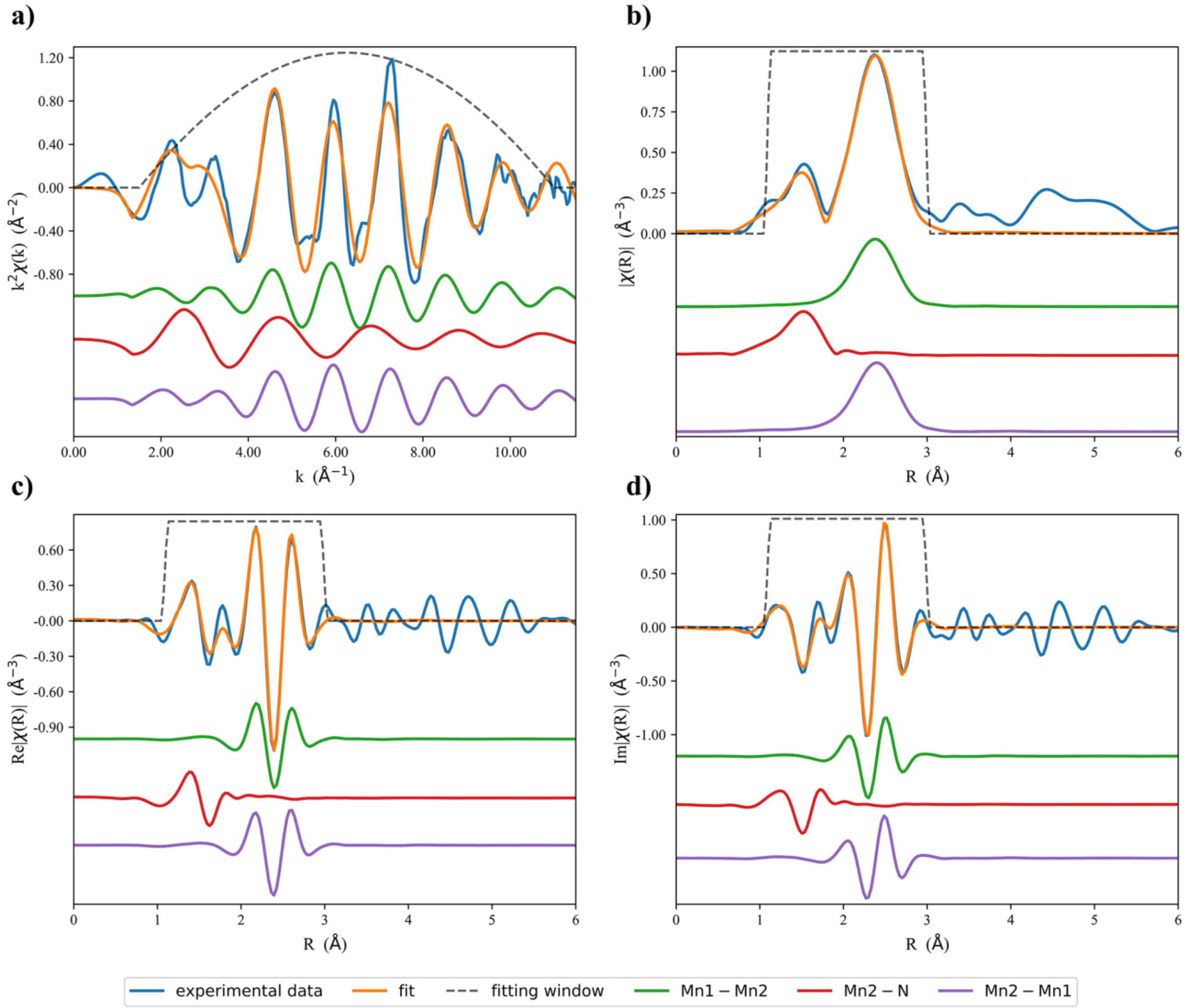

Figure S1. Fitting results of the k^2-weighted FT-EXAFS spectra of Mn_4N (Mn K-edge) shown in (a) *k*-space, (b) *R*-space magnitude, (c) *R*-space real part, and (d) *R*-space imaginary part. The *R*-space spectra are presented without phase correction. The fitting contributions of the three scattering paths considered (scaled by 0.5) are also included for reference. Additional details are provided in Table S4 and the accompanying discussion.

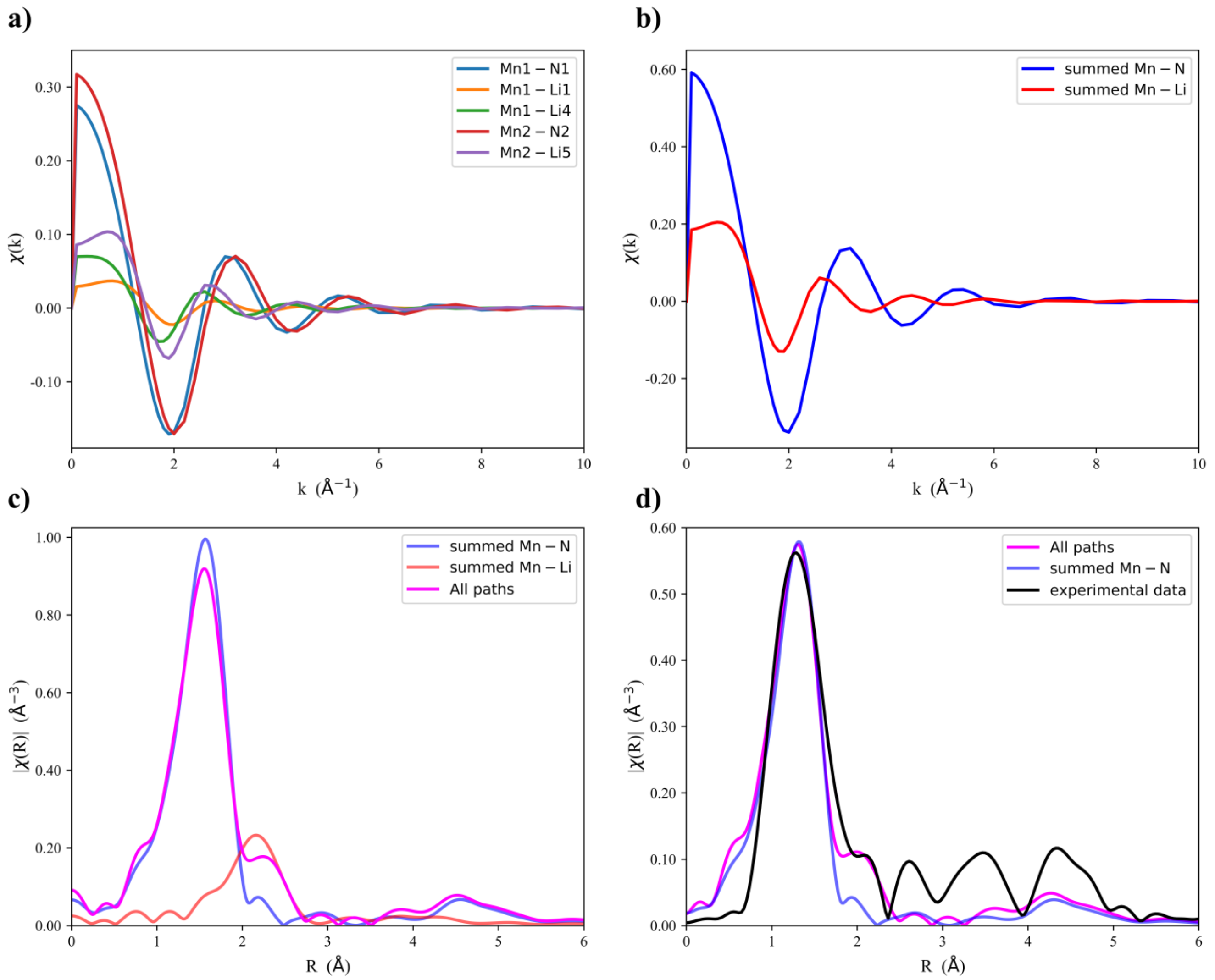

Figure S2. Results of FEFF8 calculations. (a) Individual single-scattering χ(k) functions for each path computed from the EXAFS equation. (b) Path-summed χ(k) by scatterer: summed Mn–N = Mn1–N1 + Mn2–N2; summed Mn–Li = Mn1–Li1 + Mn1–Li4 + Mn2–Li5. (c) Magnitude of the k→R Fourier transformed EXAFS, highlighting additional intensity at $R \approx 2.0$-2.5 Å from Mn–Li contributions (all paths = Mn–N + Mn–Li. (d) Comparison of calculated and experimental |χ(R)|. For visualization only (prior to fitting), the calculated traces were adjusted to facilitate qualitative comparison with the experimental data. The R-space data of summed Mn–N paths and all paths were scaled by 0.58 and 0.63, respectively, and both spectra were shifted by -0.25 Å.

Given a weak backscattering of Li due to its low atomic number, Mn–Li paths were incorporated into the EXAFS model of Li_7MnN_4 with particular caution. Prior to EXAFS fitting, FEFF8 calculations were performed using Rietveld-refined crystallographic data obtained from neutron diffraction measurements [8], where the atomic positions of Li in Li_7MnN_4 are well defined. The purpose of these calculations was to estimate the relative scattering amplitudes of Mn–N and Mn–Li paths and to evaluate the detectability of Li-related scattering within the measured *k*-range. Figure S2a shows the calculated scattering amplitudes (χ(k)) for the Mn–N and Mn–Li paths included in the FEFF calculations. These amplitudes were computed using the EXAFS equation applied to each scattering vector, as follows:

$$\chi(k) = \frac{N_j S_0^2}{kR_j^2} \cdot f_j(k) \cdot e^{\frac{-2R_j}{\lambda(k)}} \cdot e^{-2k^2\sigma_j^2} \cdot \sin[2kR_j+\delta_j(k)]$$

where *j* denotes the scatterer index (e.g., N1), *N* is the coordination number, *R* is the interatomic distance (e.g., Mn1–N1 distance), λ is the photoelectron mean free path, $f_j(k)$ is the backscattering amplitude, σ is the Debye-Waller factor, δ is the total phase shift, and S_0^2 is the amplitude reduction factor. The parameters f, λ, σ, and δ were obtained from FEFF calculations; *N* and *R* were taken from the Rietveld-refined structure, and S_0^2 was determined from the Mn_4N reference fit.

An initial glance of Figure S2a confirms that the Mn–Li scattering contributions are systematically weaker than the Mn–N vectors. Based on FEFF8 calculations, the single-scattering amplitudes for Mn1–Li1 and Mn1–Li4 are approximately 12% and 21% of Mn1–N1, respectively; for the second Mn site, Mn2–Li5 is ≈32% of Mn2–N2. In the path-summed χ(*k*) shown in Figure S2b, the Li contribution is 30-40% of Mn–N at low *k* (<2 $Å^{-1}$), falling to ≲20% by $k \approx$ 4-6 $Å^{-1}$ and becoming negligible above $k \approx 7$ $Å^{-1}$. This behavior is consistent with the low-*Z* nature of Li, whose backscattering intensity peaks at lower *k* values. The path-summed Li and N terms are largely in phase at $k \approx$ 0-1.8 $Å^{-1}$, while exhibiting a direct anti-phase relationship above $k \approx 4$ $Å^{-1}$. In the intermediate region (i.e., $k \approx$ 2-4 $Å^{-1}$), the Mn–Li contributions are neither fully cancelled nor fully reinforced by the Mn–N scattering vectors. The forward Fourier transform (*k*→*R*) shown in Figure S2c yields well-resolved and distinct Mn–N and Mn–Li peaks separated by 0.58 Å in |χ(R)| (non-phase corrected). Additionally, Figure S2c shows that the Mn–Li peak significantly exceeds the high-*R* shoulder feature of the Mn–N peak (ca. R = 2.1 Å). All of these observations indicate that Mn–Li contributions should be considered in the EXAFS fit, provided that their inclusion improves the fit statistics within the chosen fitting window (Figure S2d).

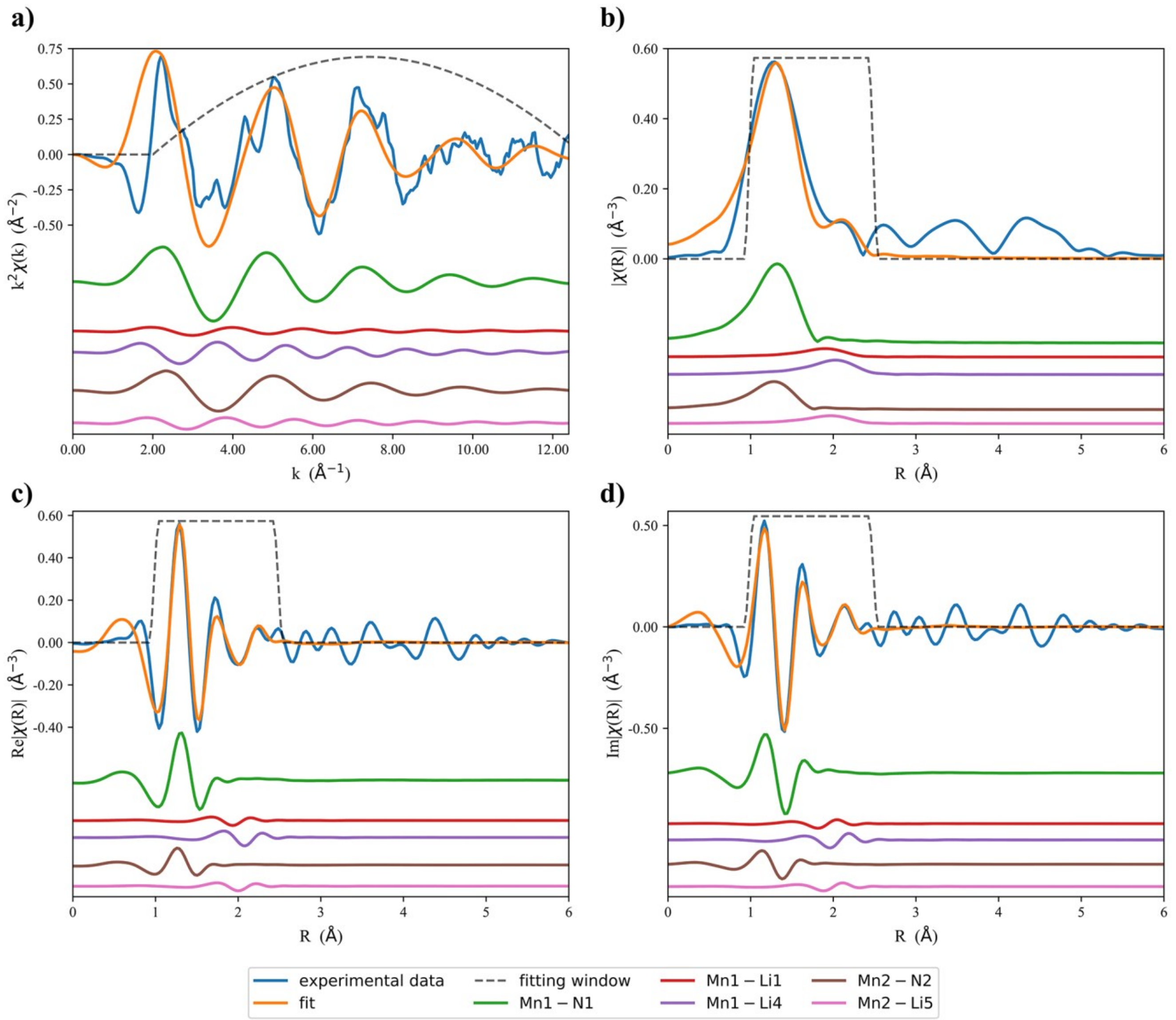

Figure S3. Fitting results of the k^2-weighted FT-EXAFS spectra of Li_7MnN_4 (Mn K-edge) shown in (a) *k*-space, (b) *R*-space magnitude, (c) *R*-space real part, and (d) *R*-space imaginary part. The *R*-space spectra are presented without phase correction. The fitting contributions of the three scattering paths considered (scaled by 0.5) are also included for reference. Additional details are provided in Table S4 and the accompanying discussion.

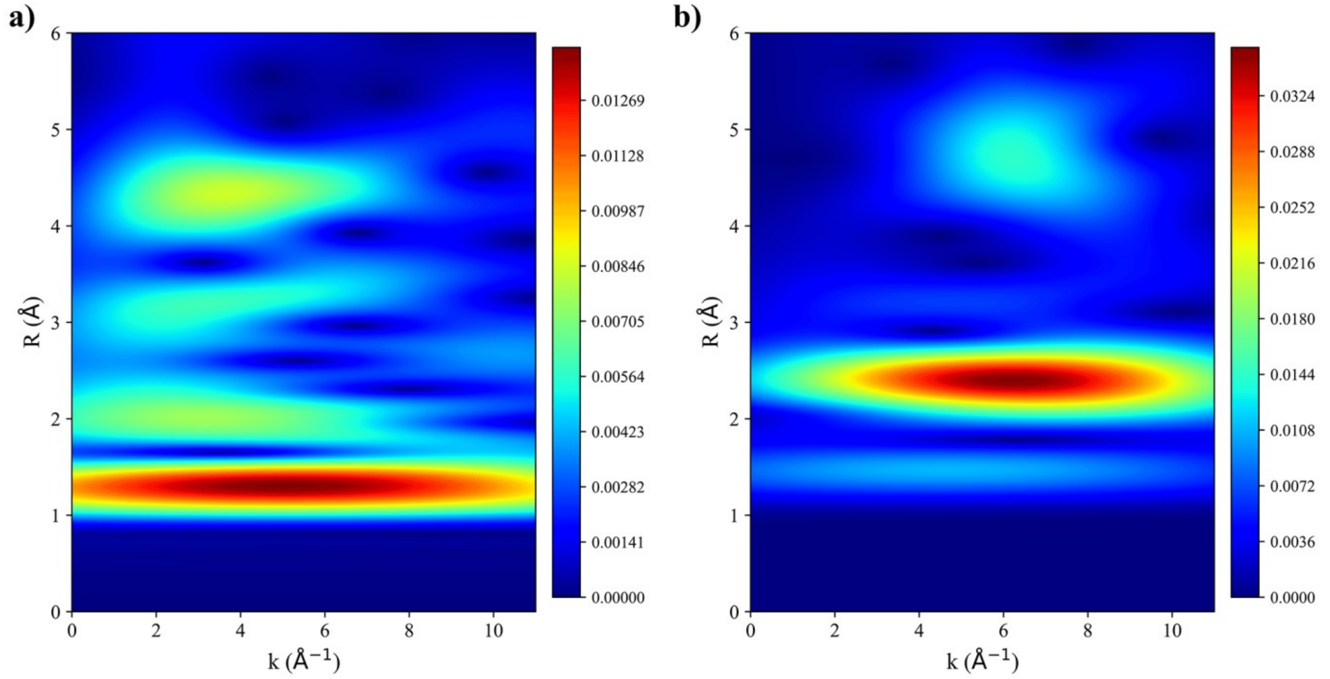

Figure S4. Wavelet transforms of the k^2-weighted EXAFS signals of Mn in Li_7MnN_4 (a) and Mn_4N (b). For Li_7MnN_4, the lobe centered at $R = 1.3$ Å arises from strong EXAFS oscillations of N scatterers within a wide k-range of 0-11 $Å^{-1}$, while the feature at $R = 2$ Å is attributed to Li scatterers within a smaller k-range of 0-7 $Å^{-1}$. This assignment to Li is consistent with FEFF8 calculations performed prior to EXAFS fitting (Figures S2a and S2b). The higher k-values observed for the first coordination shell (Mn–N) compared to the second coordination shell (Mn–Li) reflect the heavier atomic mass of N relative to Li. Features at ca. $R = 3.2$ Å and R = 4.5 Å correspond to multiple-scattering contributions involving combinations of Li, Mn, and N scatterers. For Mn_4N (panel b), the main feature at ca. $R = 2.5$ Å originates from Mn scatterers within the k-range of 2.5-11 $Å^{-1}$, while a less intense feature at $R = 1.5$ Å is attributed to N scatterers.

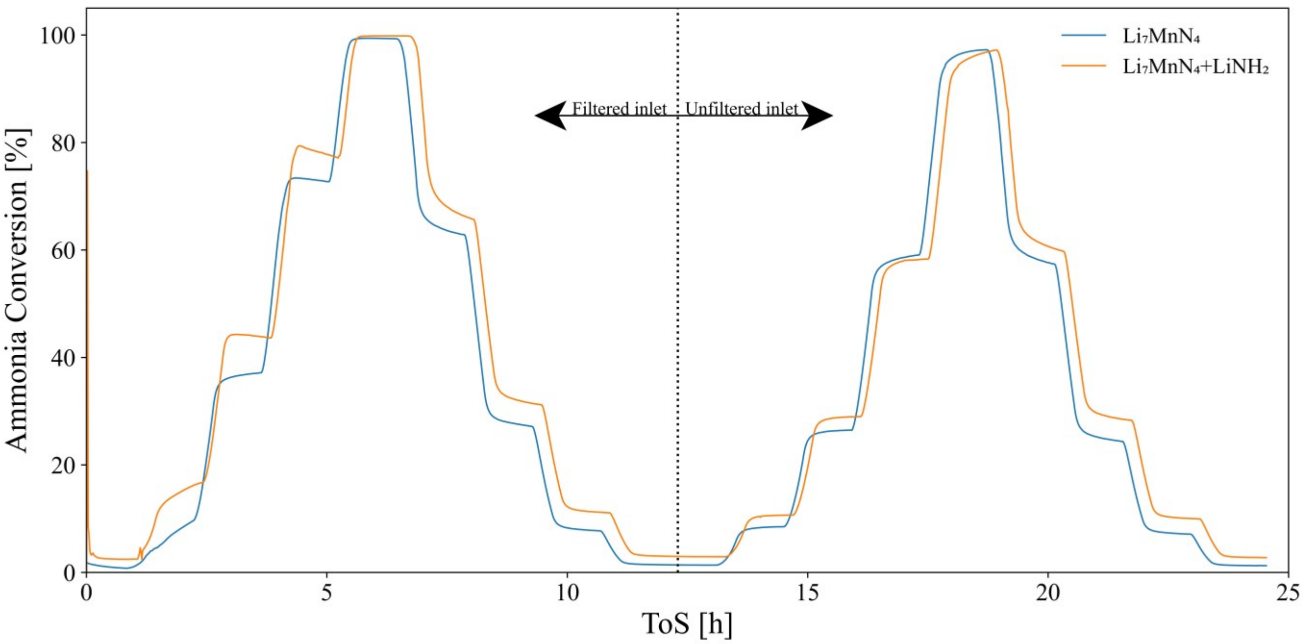

Figure S5. Time on stream of the catalytic ammonia decomposition activities of Li_7MnN_4 and Li_7MnN_4:$LiNH_2$ (1:1 molar ratio) in a temperature range between 400°C and 600°C. Reaction conditions: $P = 1$ atm, flow rate = 0.6 mL·mg_{cat}^{-1}·min^{-1}, rate of temperature changes between the steps = 2.0 K·min^{-1}.

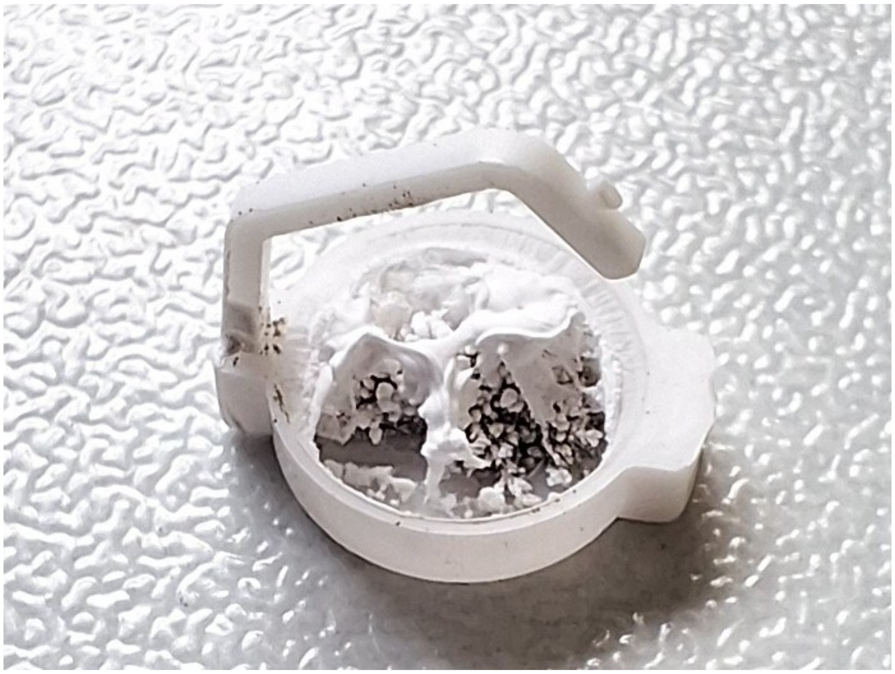

Figure S6. Digital photograph of the crucible containing the sample after the TG-MS test.

4. References

1. W. Van Beek, O.V. Safonova, G. Wiker and H. Emerich, SNBL, a Dedicated Beamline for Combined *in Situ* X-Ray Diffraction, X-Ray Absorption and Raman Scattering Experiments, *Phase Transit.*, 2011, **84**, 726–732.
2. P.M. Abdala, O.V. Safonova, G. Wiker, W. Van Beek, H. Emerich, J.A. Van Bokhoven, J. Sá, J. Szlachetko and M. Nachtegaal, Scientific Opportunities for Heterogeneous Catalysis Research at the SuperXAS and SNBL Beam Lines, *CHIMIA*, 2012, **66**, 699.
3. BM31 Swiss Norwegian Beamlines. https://www.esrf.fr/UsersAndScience/Experiments/CRG/BM01/bm01b (accessed 2025-09-04).
4. BM31 - XAS / HRPD Swiss-Norwegian Beamline BM31. https://www.wayforlight.eu/beamline/23262 (accessed 2025-09-04).
5. User Beam Mode Schedule 2025/I, 2025. https://www.esrf.fr/files/live/sites/www/files/Accelerators/Operation/Modes/Sched%202025-I%20User%20modes%20Final%2020Jan2025.pdf (accessed 2025-08-26).
6. M. Newville, Larch: An Analysis Package for XAFS and Related Spectroscopies, *J. Phys. Conf. Ser.*, 2013, **430**, 012007.
7. M. Muñoz, P. Argoul and F. Farges, Continuous Cauchy Wavelet Transform Analyses of EXAFS Spectra: A Qualitative Approach, *Am. Mineral.*, 2003, **88**, 694–700.
8. J. Cabana, N. Dupré, G. Rousse, C.P. Grey, M.R. Palacín, Ex situ NMR and neutron diffraction study of structure and lithium motion in Li_7MnN_4, *Solid State Ion.*, 2005, **176**, 2205–2218.
9. R. Niewa, F.R. Wagner, W. Schnelle, O. Hochrein, R. Kniep, $Li_{24}[MnN_3]_3N_2$ and $Li_5[(Li_{1-x}Mn_x)N]_3$, Two Intermediates in the Decomposition Path of $Li_7[MnN_4]$ to $Li_2[(Li_{1-x}Mn_x)N]$: An Experimental and Theoretical Study, *Inorg. Chem.*, 2001, **40**, 5215–5222.
10. R. Yu, X. Chong, Y. Jiang, R. Zhou, W. Yuan and J. Feng, The Stability, Electronic Structure, Elastic and Metallic Properties of Manganese Nitrides, *RSC Adv.*, 2015, **5**, 1620–1627.
